# Distribution of major lymphocyte subsets and memory T-cell subpopulations in healthy adults employing GLP-conforming multicolor flow cytometry

**DOI:** 10.1038/s41375-021-01348-5

**Published:** 2021-07-21

**Authors:** Christian R. Schultze-Florey, Ekaterina Chukhno, Lilia Goudeva, Rainer Blasczyk, Arnold Ganser, Immo Prinz, Reinhold Förster, Christian Koenecke, Ivan Odak

**Affiliations:** 1 Institute of Immunology, Hannover Medical School, Hannover, Germany; 2 Department of Hematology, Hemostasis, Oncology and Stem Cell Transplantation, Hannover Medical School, Hannover, Germany; 3 Institute of Transfusion Medicine and Transplant Engineering, Hannover Medical School, Hannover, Germany; 4 Cluster of Excellence RESIST (EXC 2155), Hannover Medical School, Hannover, Germany; 5 German Centre for Infection Research (DZIF), Partner site, Hannover, Germany

**Keywords:** Lymphocytes, Medical research

## To the Editor:

Complex flow cytometry is widely used in a broad variety of indications for prognosis, diagnosis, and therapy assessment of malignancies, infectious diseases, and immunodeficiencies [1]. In order to provide valid interpretation of flow data, comparison with reference values generated from age- and sex-matched healthy populations is needed.

Significant amount of research has been done to establish reference values of immune cells in healthy donors [2–4]. However, combining those studies into one comprehensive data set is an error-prone task due to variable limitations of these studies: some studies covered only a narrow range of leukocyte subsets analyzed due to low numbers of monoclonal antibodies (mAb) applied [5], while others included only limited numbers of samples for analysis. Furthermore, some studies primarily compared young and old age groups, or lacked the full age coverage. Another limitation of several studies is the lack of separation of γδ from αβ T cells [6]. This distinction would be relevant since there is increasing evidence that γδ T cells play an important role in immunity, cancer, and infections [7]. While Ballester et al. provided reference values for bulk γδ T cells, no reference values for the clinically relevant Vγ9^+^ subpopulation are provided in that analysis [8]. A recent study [9] has advanced the field by providing reference values for B cells, NK cells, granulocytes and monocytes, but segregation into biologically relevant age groups is still under debate. A recent publication described significant changes in the transcriptome of white blood cells occurring at the age of 40 years and shortly after the age of 60 years [10], raising the need for new age-dependent reference values, as age categories had mostly been defined arbitrarily or solely based on cohort proportions. Moreover, there is a major lack of reference values regarding in-depth profiling of antigen-experienced lymphocytes. Here, CD45RA and CD62L are commonly used markers to discriminate T cells based on their antigen memory phenotype [11]. We and others have shown earlier that higher numbers of antigen-experienced cells are associated with clearance of infection [12, 13]. Hence, standardized reference values for lymphocyte memory subpopulations are a prerequisite for data interpretation in clinical settings. All in all, considering the advances in complex flow cytometry for immune profiling, a set of current reference values of detailed lymphocyte subsets is largely missing. In this study, we used two standardized, Good Laboratory Practice (GLP)-conforming flow cytometry panels to establish reference values for 12 major lymphocyte subsets, and additional 16 memory subsets of αβ CD4^+^_conv_, CD8^+^, γδ, and regulatory T cells (T_regs_) in peripheral blood in a cohort of 244 healthy adults. We provide frequencies and absolute values based on certified bead count technology and stratified data based on age and sex categories.

In the present study, a total of 244 healthy adults were recruited at Hannover Medical School between October 28th and November 27th, 2019. Additional 22 volunteers were recruited for the purpose of panel validation prior to October 28th 2019. The study was approved by the local institutional review board (#8606_BO_K_2019) and informed consent was obtained from all participants.

All samples were processed and, as described in the Supplementary Data, stained with two panels of mAb (panels 1 and 2; Supplementary Table 1). Both panels were validated according to German Industrial Norm (DIN) DIN EN ISO 15189:2014 prior to the study and thus concur with GLP criteria. Detailed description of the panel validation process is given in Supplementary Data (Supplementary Tables 2–6).

In panel 1, mAb together with TruCount™ beads (BD Biosciences, San Jose, CA, USA) were used to analyze 12 major lymphocyte populations (Supplementary Fig. 1A). Additional 16 memory populations were assessed using panel 2 mAb (Supplementary Fig. 1B), and absolute numbers were calculated based on the absolute values of CD4_conv_ and CD8^+^ from panel 1. Importantly, although different processing techniques were applied for panels 1 and 2 (Supplementary Materials and Methods), we observed no difference regarding the frequencies of the major lymphocyte subsets (Supplementary Fig. 2) nor their respective memory subpopulations (Supplementary Fig. 3) when sample processing techniques were interchanged.

Blood samples from 244 healthy adults were analyzed. 120 of them were from females (49.2%) and 124 were from males (50.8%). The age range was 18–69 years and the average age for females was 39 years and for males 43 years of age. Based on recent transcriptomic data addressing age-dependent effects on immune cell subpopulations [10], the cohort analyzed in the present study was split into two groups: young (18–40 years: 61 females, 52 males) and middle-aged (41–69 years: 59 females, 72 males) participants. Age was evenly distributed across both female and male subgroups (Supplementary Fig. 4A). All reference values are given in Table 1 (absolute numbers) and Supplementary Table 7 (frequencies). Moreover, complete blood counts were performed (Supplementary Table 8).

**Table 1:** Reference values of lymphocyte populations including memory values in healthy adults.

Absolute counts of lymphocyte subsets								
Males					Females			
	18–40 years old		41–69 years old		18–40 years old		41–69 years old	
CD4_conv_	623.1	(286.3–1228.0)	495.6	(179.7–1109.0)	858.3	(376.7–6027)	607.7	(232.8–2227)
T_regs_	73.9	(27.8–166.4)	71.3	(36.8–185.8)	75.6	(22.2–410.7)	73.6	(20.6–163.5)
CD8^+^CD4^+^	4.7	(1.5–40.7)	5.2	(1.2–38.4)	6.6	(2.3–40.6)	7.2	(0.8–25.2)
CD8^+^	393.0	(202.1–907.7)	266.8	(89.8–799.3)	419.9	(196.6–4100.0)	271.8	(137.8–630.4)
γδ T cells	59.3	(13.5–473.3)	30.3	(4.8–194.3)	66.2	(16.5–749.4)	32.9	(4.7–180.5)
Vγ9^+^	38.6	(3.9–464.2)	15.7	(2.3–120.9)	47.9	(8.5–568.3)	20.8	(1.3–119.0)
Vγ9^−^	13.8	(2.5–113.5)	6.5	(1.2–92.7)	15.2	(2.6–170.6)	7.1	(1.5–106.3)
B cells	231.3	(88.5–500.5)	226.7	(86.9–503.5)	271.6	(109.4–1834.0)	213.1	(74.3–633.6)
CD20^+^	220.6	(79.7–484.6)	215.9	(80.2–473)	266.7	(98.8–1805.0)	204.4	(69.2–627.0)
CD20^−^	6.6	(3.1–18.5)	5.9	(3.3–43.7)	5.00	(2.3–26.6)	4.6	(1.4–13.7)
NK cells	163.5	(41.3–545.4)	189.6	(57.5–611.1)	217.9	(28.8–839.3)	171.3	(60.9–423.1)
NKT cells	48.1	(13.8–276.6)	32.3	(6.2–503.5)	55.2	(11.6–384.3)	42.3	(7.6–200.0)
CD4conv								
naive	352.9	(110.8–777.2)	201.1	(46.6–553.1)	498.4	(145.5–3184.0)	300.8	(60.1–1709.0)
cm	184.4	(1.6–339.4)	155.6	(60.0–363.1)	235.7	(8.0–2129.0)	187.5	(69.1–535.5)
em	93.8	(1.3–197.1)	84.4	(33.7–268.6)	121.6	(5.0–678)	95.2	(46.8–203.6)
temra	7.3	(1.4–137.5)	6.3	(1.0–44.4)	7.9	(0.7–166.3)	8.0	(2.1–37.6)
Tregs								
naive	33.1	(9.9–82.1)	19.6	(3.7–48.6)	32.1	(9.2–220.8)	19.9	(4.0–64.1)
cm	33.3	(1.0–76.9)	46.1	(19.6–118.0)	36.4	(3.3–175.9)	43.6	(12.3–106.5)
em	4.2	(0.4–13.2)	5.7	(2.0–18.5)	4.4	(0.9–29.4)	5.3	(2.0–13.5)
temra	0.3	(0.1–6.4)	0.2	(0.0–0.9)	0.3	(0.0–9.3)	0.2	(0.0–1.5)
CD8								
naive	159.6	(58.0–455.9)	69.4	(19.7–192.2)	227.6	(59.6–2431.0)	91.8	(28.6–297.7)
cm	68.0	(10.7–169.1)	58.7	(13.5–262.6)	67.0	(18.6–437.4)	54.8	(22.1–183.4)
em	26.8	(0.1–133.1)	24.7	(6.8–153.1)	24.2	(0.1–471.1)	24.9	(6.3–73.9)
temra	104.6	(29.0–385.8)	69.3	(23.3–390.4)	79.9	(29.7–883.2)	75.2	(18.1–322.3)
γδ T cells								
naïve-like	6.8	(1.1–218.2)	1.7	(0.3–19.1)	9.0	(1.5–129.5)	3.3	(0.6–25.3)
cm-like	24.5	(0.1–209.1)	9.3	(1.7–92.8)	33.1	(0.5–498.9)	13.0	(0.9–72.3)
em-like	11.2	(0.2–134.7)	7.1	(1.3–53.0)	11.3	(0.4–105.2)	5.9	(0.8–81.1)
temra-like	6.0	(0.5–90.3)	3.5	(0.5–84.0)	3.7	(0.3–98.1)	4.2	(0.3–93.7)
	*N* = 52		*N* = 72		*N* = 60		*N* = 60	

Data are presented as median (2.5–97.5%) percentile. Values are expressed as per µl of blood.

**Fig. 1:**
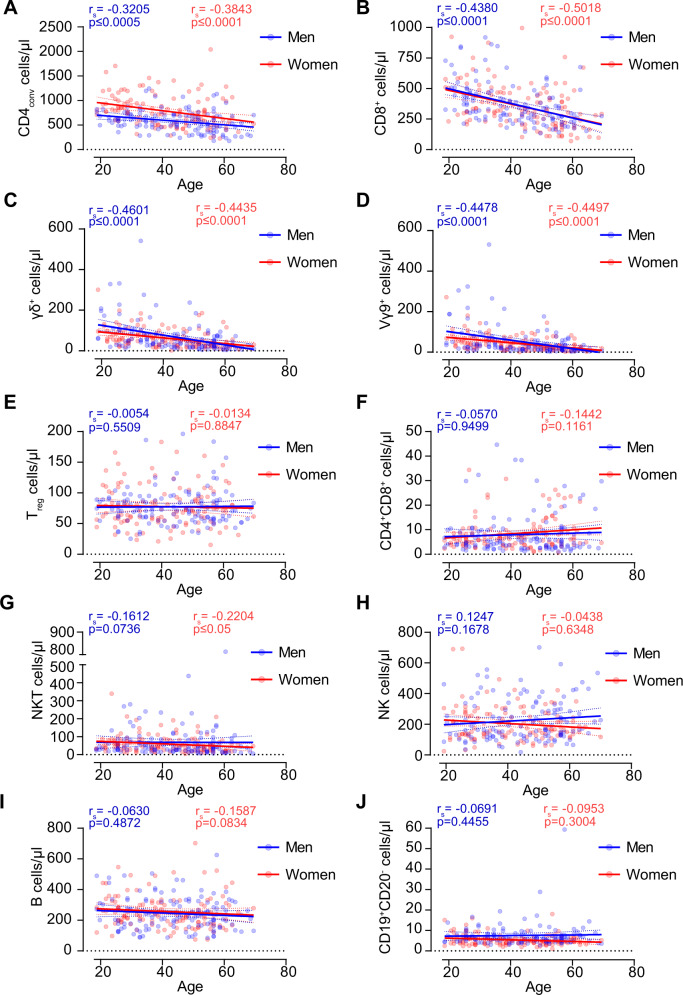
Age effect on absolute cell counts of lymphocyte populations. Lines represent linear regression. Dotted lines represent 95% confidence intervals for the linear regression line. Each dot represents an individual sample. Statistical analysis was performed by Spearman’s correlation test. *r*_*s*_ = Spearman rank correlation coefficient.

First, we compared the general frequency and numbers of lymphocyte amongst all white blood cells between sexes. We observed a marginal increase in frequencies and numbers of lymphocytes and leukocytes in women compared to men (Supplementary Fig. 4B, C). Next, we compared the numbers of 12 lymphocyte subsets in men and women and only found significantly higher absolute numbers of CD4_conv_ cells in women (Supplementary Fig. 4D). In addition, frequencies of CD4_conv_ cells were also increased significantly in women whilst frequencies of regulatory T, NK, and CD19^+^CD20^−^ B cells were significantly increased in males (Supplementary Fig. 5). Next, we analyzed the impact of age on lymphocyte numbers. We detected a significant reduction of CD4_conv_ with advancing age in both women and men (Fig. 1A). A similar but markedly stronger reduction effect was observed in the CD8^+^ T-cell compartment, also both in women and men (Fig. 1B). Furthermore, γδ T cells, including the Vγ9^+^ fraction within this population, were also inversely correlated with age (Figs. 1C, D). Exceptions amongst the T-cell compartment were T_reg_ cells and CD4^+^CD8^+^ T cells where we observed no impact of age on cell counts (Figs. 1E, F). Of note, we detected a minor decrease in NKT cells with age (Fig. 1G). This finding however was only observed in women. No correlation was found between age and cell numbers for NK cells (Fig. 1H), CD19^+^ B cells (Fig. 1I) and the CD19^+^CD20^−^ B-cell subset (Fig. 1J).

Based on previous antigen experience, CD4_conv_ cells and CD8^+^ αβ T cells are classically addressed as naive (T_naive_), terminally differentiated effector memory (T_temra_), effector memory (T_em_), and central memory (T_cm_) cells [11, 14, 15]. To test for a possible effect of age on the distribution of memory lymphocyte subsets, we correlated the frequency of the different lymphocyte subpopulations in our cohort with age. Out of the major T-cell subsets, we noticed a pronounced reduction in frequencies of CD4_naive_ cells and a corresponding increase of CD4_temra_, CD4_em_, and CD4_cm_ cells in women (Supplementary Fig. 6A) and men (Supplementary Fig. 7A). A decrease of CD4_naive_ cells, but not memory subsets cells were also observed in absolute cell counts (Supplementary Figs. 6B and  7B). Similar, but more pronounced effects were also observed in CD8^+^ memory subsets (Supplementary Figs. 6C, D and 7C, D). The effect of age on the distribution of T-cell subtypes was, however, most pronounced within the T_reg_ compartment. While naïve T_reg_ cells inversely correlated with age in both frequencies and numbers, we observed a strong positive correlation of T_cm_–T_reg_ cells with age (Supplementary Figs. 6E, F and 7E, F). Furthermore, both naïve-like and T_cm_-like γδ T cells inversely correlated with age in both frequencies and numbers, whereas T_temra_-like γδ T cells correlated in frequencies, but not in numbers, with age (Supplementary Figs. 6G, H and 7G, H).

In this study, we used two state-of-the-art clinical grade GLP-conforming staining panels to establish reference intervals for 12 major lymphocyte subsets and 16 memory subpopulations in a large cohort of non-elderly healthy adults. The general frequencies and absolute numbers of B and T cells in our cohort were comparable with the ranges described in previous publications [2–6, 9], confirming the validity of our data. Following recent findings on age-dependent transcriptional changes in immune cells [10], we allocated 28 lymphocyte subpopulations in frequencies and absolute numbers to young (18–40 years) and middle-aged (41–69 years) adults. Employing these transcriptional-based age categories, we observed decreasing numbers of circulating CD4_conv_, CD8^+^, and γδ T cells with age both in men and women (Fig. 1) as well as a shift from naive T cells toward cells showing central memory and effector memory phenotypes in the middle-aged subgroup (Supplementary Figs. 6 and 7). A clear limitation of our study is the lack of elderly healthy adults (70 years and older) and the lack of delineation of early, mature, and terminally differentiated NK cells. Thus, future studies should also assess lymphocyte subpopulation changes in elderly healthy adults and establish reference values for NK cell subsets. Moreover, inclusion of a myeloid cell marker could further increase the purity of the lymphocyte population. Taken together, we provide age- and sex-specific ranges of frequencies and absolute numbers of immunological relevant subpopulations in a large cohort of healthy adults.

## Supplementary information

Supplemental Data- clean

Supplementary Figure 1

Supplementary Figure 2

Supplementary Figure 3

Supplementary Figure 4

Supplementary Figure 5

Supplementary Figure 6

Supplementary Figure 7

## References

[1] Vembadi A, Menachery A, Qasaimeh MA (2019). Cell cytometry: review and perspective on biotechnological advances. Front Bioeng Biotechnol.

[2] Whitby L, Whitby A, Fletcher M, Helbert M, Reilly JT, Barnett D (2013). Comparison of methodological data measurement limits in CD4+ T lymphocyte flow cytometric enumeration and their clinical impact on HIV management. Cytom Part B - Clin Cytom.

[3] Böhm I (2006). Quantification of absolute peripheral white blood cells and their subsets in patients with lupus erythematosus: comparison with other inflammatory diseases with and without autoimmune background. Biomed Pharmacother.

[4] Andreu-Ballester JC, García-Ballesteros C, Benet-Campos C, Amigó V, Almela-Quilis A, Mayans J, et al. Values for αβ and γδ T-lymphocytes and CD4+, CD8+, and CD56+ subsets in healthy adult subjects: assessment by age and gender. Cytom Part B Clin Cytom. 2012;82B:238–44. Available from: 10.1002/cyto.b.21020. 10.1002/cyto.b.21020 22539222

[5] Uppal SS, Verma S, Dhot PS (2003). Normal values of CD4 and CD8 lymphocyte subsets in healthy indian adults and the effects of sex, age, ethnicity, and smoking. Cytometry.

[6] Mund E, Christensson B, Larsson K, Grönneberg R (2001). Sex dependent differences in physiological ageing in the immune system of lower airways in healthy non-smoking volunteers: study of lymphocyte subsets in bronchoalveolar lavage fluid and blood. Thorax.

[7] Hayday AC (2019). γδ T cell update: adaptate orchestrators of immune surveillance. J Immunol.

[8] Nussbaumer O, Thurnher M (2020). Functional phenotypes of human Vγ9Vδ2 T cells in lymphoid stress surveillance. Cells.

[9] Melzer S, Zachariae S, Bocsi J, Engel C, Löffler M, Tárnok A (2015). Reference intervals for leukocyte subsets in adults: Results from a population-based study using 10-color flow cytometry. Cytom Part B - Clin Cytom.

[10] Márquez EJ, Chung C han, Marches R, Rossi RJ, Nehar-Belaid D, Eroglu A, et al. Sexual-dimorphism in human immune system aging. Nat Commun. 2020;11. Available from: 10.1038/s41467-020-14396-9. 10.1038/s41467-020-14396-9 PMC7005316 32029736

[11] Lei H, Kuchenbecker L, Streitz M, Sawitzki B, Vogt K, Landwehr-Kenzel S (2015). Human CD45RA-FoxP3hi memory-type regulatory T cells show distinct TCR repertoires with conventional T cells and play an important role in controlling early immune activation. Am J Transplant.

[12] Odak I, Barros-Martins J, Bošnjak B, Stahl K, David S, Wiesner O, et al. Reappearance of effector T cells is associated with recovery from COVID-19. EBioMedicine. 2020;57:102885. Available from: https://linkinghub.elsevier.com/retrieve/pii/S2352396420302607. 10.1016/j.ebiom.2020.102885 PMC7338277 32650275

[13] Sattler A, Angermair S, Stockmann H, Heim KM, Khadzhynov D, Treskatsch S, et al. SARS-CoV-2 specific T-cell responses and correlations with COVID-19 patient predisposition. J Clin Invest. 2020. Available from: http://www.ncbi.nlm.nih.gov/pubmed/32833687. 10.1172/JCI140965 PMC7685725 32833687

[14] Martin MD, Badovinac VP (2018). Defining memory CD8 T cell. Front Immunol.

[15] Hengel RL, Thaker V, Pavlick MV, Metcalf JA, Dennis G, Yang J (2003). Cutting edge: L-selectin (CD62L) expression distinguishes small resting memory CD4 + T cells that preferentially respond to recall antigen. J Immunol.

