# Supplementary material for: Distribution of major lymphocyte subsets and memory T-cell subpopulations in healthy adults employing GLP-conforming multicolor flow cytometry
Source: Leukemia. 2021 Jul 21;35(10):3021–5. doi: 10.1038/s41375-021-01348-5 (PMC8478656; doi:10.1038/s41375-021-01348-5)
Supplement: Supplementary file 1 — Supplemental Data- clean [file 41375_2021_1348_MOESM1_ESM.docx]

**Supplementary Data**

**Supplementary Materials and Methods**

*Panel validation process*

To validate both panels, we tested i) stability of the antibody mastermix (Supplementary Table 2A, 2B), ii) sample stability prior to staining (Supplementary Table 3A, 3B), iii) panels diagnostic thresholds (Supplementary Table 4A, 4B) and iv) panels precision (Supplementary Table 5A, 5B). Moreover, to test the measurement precision, we used samples from four volunteers treated with rituximab for hematological malignancies, which allowed us to evaluate the B cells as a known negative parameter. Data obtained from those 4 volunteers with both panels on the BD^TM^ FACS Lyric were compared to a commercially available 6-color TBNK BD^TM^ panel measured on a BD^TM^ FACSCanto II (Supplementary Table 6A). In addition, we compared the lymphocyte count precision against the Sysmex XN-9100^TM^ (Sysmex, Kobe, Japan) hemocytometer (Supplementary Table 6B).

*Sample preparation and acquisition*

For panel 1 100µl EDTA blood was inversely pipetted into the BDTrucount^TM^ tube together with 10µl of antibody mastermix (Supplementary Table 1) followed by brief vortexing. After 15 minutes incubation at room temperature in the dark 1000µl of 1:10 diluted BD^TM^ FACS lysing solution was added and vortexed. After another incubation step of 15 minutes at room temperature in the dark the tube was measured (lyse-no-wash). For panel 2, 100µl EDTA blood was pipetted into a tube together with 30µl of antibody mastermix (Supplementary Table 1), followed by brief vortexing. After 15 minutes incubation at room temperature in the dark 2000µl of 0.83% ammonium chloride lysing solution was added and vortexed. After another incubation step of 15 minutes at room temperature in the dark, the tube was centrifuged at 311g for 5 minutes. The supernatant was discarded and the pellet was washed with 2000µl PBS. After another centrifugation step at 2000rpm for 5 minutes followed by discarding of the supernatant the pellet was resuspended in 200µl PBS and measured (lyse-wash protocol). All samples were processed and acquired in an immediate and sequential order. For panel 1, acquisition was stopped automatically after 50 000 singlet lymphocyte events (SSC-W/SSC-H plot in the Supplementary Figure 1A) or 180 seconds (whatever was reached first). For panel 2, acquisition was stopped automatically after 50 000 singlet viable lymphocyte events (7-AAD/SSC-H plot in Supplementary Figure 1B) or 120 seconds (whatever was reached first). The minimal diagnostic threshold for stable, reproducible results was set at 2000 lymphocyte events for panel 1 and 6000 viable lymphocytes for panel 2 based on the dilution experiments within the DIN EN ISO 15189:2014 validation process (Supplementary Table 4A, 4B). All samples were acquired on BD^TM^ FACS Lyric flow cytometer. In this study no sample was in the acquisition queue after staining for longer than 120 min.

Quality control measures were in place, requiring daily calibration of the BD^TM^ FACS Lyric flow cytometer using BD^TM^ CS&T Beads as well as weekly measurements of BD^TM^ multi-check control (control blood). Passing these quality control measures is a prerequisite for measurement of lymphocyte subsets with panel 1 and 2.

*Data analysis*

Flow cytometry data were analyzed using FCS Express 7 (De Novo Software, Pasadena, CA, USA). Gates were set manually and reliability of gating for each sample was assessed independently by two evaluators. Statistical analysis was performed with Prism 8 (GraphPad, San Diego, CA, USA) and Gpower 3.1. (Erdfelder, Faul, & Buchner, 1996)

**Supplementary Figure Legends**

**Supplementary Figure 1:** Gating strategies. (A) Gating strategy for the analysis of absolute cell counts. (B) Gating strategy for the analysis of memory populations. Arrows indicate gating hierarchy.

**Supplementary Figure 2**: Accuracy testing for panels 1 and 2 used with lyse-no-wash (LnW) or lyse-wash (LW) protocol. N=20 healthy volunteers. Histograms and bars represent mean ± SD. All data was tested for normal distribution. Statistical significance was determined with one-way ANOVA or Kruskal-Wallis according to data normality distribution. Differences in frequencies of Vγ9^+/-^ populations and CD20^+/-^ populations were measured by Mann-Whitney U-test. No statistically significant differences were observed.

**Supplementary Figure 3**: Comparison of panel 2 mAbs used with lyse-no-wash (LnW) or lyse-wash (LW) protocol. N=20 healthy volunteers. Histograms and bars represent mean + SD. All data was tested for normal distribution. Statistical significance was determined with one-way ANOVA or Kruskal-Wallis according to data normality distribution. No statistically significant differences were observed.

**Supplementary Figure 4**: Absolute cell counts of lymphocyte populations in healthy men and women. (A) Age distribution of healthy volunteers. (B) Lymphocyte frequencies and absolute counts numbers per µl of blood. (C) Absolute leukocyte counts per µl of blood. (D) Absolute cell counts of lymphocyte subpopulations. Each dot represents an individual sample. Lines represent median. Statistical analysis was performed by Student’s t-test or Mann-Whitney U test with Wilcoxon-Mann-Whitney post-hoc test where applicable. d=effect size; 1-β= power; **p≤0.01; ****p≤0.0001

**Supplementary Figure 5:** Frequencies of lymphocyte populations in healthy men and women. Each dot represents an individual sample. Lines represent median. Statistical analysis was performed by Student’s t-test or Mann-Whitney U test with Wilcoxon-Mann-Whitney post-hoc test where applicable. d=effect size; 1-β= power; *p≤0.05 **p≤0.01 ****p≤0.0001

**Supplementary Figure 6:** Age affects memory distribution of T cell subsets in women. Lines represent linear regression. Dotted lines represent 95% confidence intervals for the linear regression line. Each dot represents an individual sample. Statistical analysis was performed by Spearman’s correlation test. r_s_= Spearman rank correlation coefficient.

**Supplementary Figure 7:** Age effects memory distribution of T cell subsets in men.

Lines represent linear regression. Dotted lines represent 95% confidence intervals for the linear regression line. Each dot represents an individual sample. Statistical analysis was performed by Spearman’s correlation test. r_s_= Spearman rank correlation coefficient.

| **Panel 1** |  |  |  |  |  |  |
| --- | --- | --- | --- | --- | --- | --- |
| **Antigen** | **Flurochrome** | **RRID** | **Cat-No** | **Lot-No** | **Clone** | **Dilution** |
| CD3 | APC | AB_398591 | 555335 | 8316946 | UCHT1 | 1:200 |
| CD4 | FITC | Not listed | 345768 | 9008510 | SK3 | 1:200 |
| CD8 | APC-H7 | AB_1645736 | 641400 | 9225077 | SK1 | 1:200 |
| CD19 | PE | AB_395813 | 555413 | 8235921 | HIB19 | 1:50 |
| CD20 | BV605 | Not listed | 747736 | 9308431 | 2H7 | 1:200 |
| CD25 | BV421 | AB_11154578 | 562442 | 9095648 | M-A251 | 1:100 |
| CD45 | PerCP | Not listed | 345809 | 9007823 | 2D1 | 1:200 |
| CD56 | PE-Cy7 | AB_2857328 | 335826 | 9196215 | NCAM16.2 | 1:100 |
| CD127 | APC-R700 | AB_2739099 | 565185 | 9220640 | HIL-7R-M21 | 1:100 |
| TCRgd | PE | Not listed | 333141 | 9191485 | 11F2 | 1:200 |
| Vg9 | BV510 | AB_2741945 | 744035 | 9086901 | B3 | 1:200 |
| BD FACS™ Lysing Solution |  | Not listed | 349202 | 9141933 |  |  |
| BD Trucount™ Tubes |  | Not listed | 340334 | 19207 |  |  |
| **Panel 2** |  |  |  |  |  |  |
| CD3 | APC | AB_398591 | 555335 | 8316946 | UCHT1 | 1:200 |
| CD4 | FITC | Not listed | 345768 | 9008510 | SK3 | 1:200 |
| CD8 | APC-H7 | AB_1645736 | 641400 | 9225077 | SK1 | 1:200 |
| CD19 | PE | AB_395813 | 555413 | 8235921 | HIB19 | 1:50 |
| CD25 | BV421 | AB_11154578 | 562442 | 9095648 | M-A251 | 1:100 |
| CD45RA | BV510 | AB_2722499 | 563031 | 9014627 | HI100 | 1:200 |
| CD56 | PE-Cy7 | AB_2857328 | 335826 | 9196215 | NCAM16.2 | 1:100 |
| CD62L | BV605 | AB_2744441 | 562719 | 8173613 | DREG-56 | 1:100 |
| CD127 | APC-R700 | AB_2739099 | 565185 | 9220640 | HIL-7R-M21 | 1:100 |
| TCRgd | PE | Not listed | 333141 | 9191485 | 11F2 | 1:200 |
| ViaProbe | PerCP | Not listed | 555816 | 9052709 |  | 1:10 |
| Ammonium Chloride Lysis |  |  | House made |  |  | 0.83% |

**Supplementary Table 1: Antibodies and Reagents.**

|  | **Material** | **Lot number** | **Lymph abs (cells/µl)** | **CD3 %L** | **CD3/µl** | **CD4 %L** | **CD4/µl** | **CD8 %L** | **CD8/µl** | **CD56 %L** | **CD56/µl** | **CD19 %L** | **CD19/µl** | **CD20 %L** |  |  |  |  |  |
| --- | --- | --- | --- | --- | --- | --- | --- | --- | --- | --- | --- | --- | --- | --- | --- | --- | --- | --- | --- |
| BD multi-check control (Reference) | BD multi-check control | BM0419N | 1602 | 72.3  (62.3-82.3) | 1158.2 (926.6-1389.9) | 46.7  (40.2-53.2) | 748.1 (598.5-897.8) | 23.0  (16.0-30.0) | 368.5 (265.7-471.3) | 10.9  (3.9-17.9) | 174.6  (92.5-256.7) | 15.3  (11.3-19.3) | 245.1 (169.6-320.6) | 16.0  (11.0-21.0) |  |  |  |  |  |
|  |  |  |  |  |  |  |  |  |  |  |  |  |  |  |  |  |  |  |  |
| Mastermix  1 day old | BD multi-check control | BM0419N | 1532.30 | 73.81 | 1130.90 | 45.33 | 694.60 | 20.76 | 318.10 | 9.82 | 150.5 | 15.52 | 237.8 | 13.87 |  |  |  |  |  |
| Mastermix  1 month old | BD multi-check control | BM0419N | 1617.00 | 70.79 | 1144.60 | 44.15 | 713.80 | 19.52 | 315.70 | 10.79 | 174.4 | 17.66 | 285.6 | 14.37 |  |  |  |  |  |
|  |  |  |  |  |  |  |  |  |  |  |  |  |  |  |  |  |  |  |  |
| Average deviation |  |  | 42.35 | 1.51 | 6.85 | 0.59 | 9.60 | 0.62 | 1.20 | 0.485 | 11.95 | 1.07 | 23.9 | 0.25 |  |  |  |  |  |
| Mean |  |  | 1574.65 | 72.30 | 1137.75 | 44.74 | 704.20 | 20.14 | 316.90 | 10.305 | 162.45 | 16.59 | 261.7 | 14.12 |  |  |  |  |  |
| Standard deviation |  |  | 42.35 | 1.51 | 6.85 | 0.59 | 9.60 | 0.62 | 1.20 | 0.48 | 11.95 | 1.07 | 23.90 | 0.25 |  |  |  |  |  |
| Variationscoefficient % |  |  | 2.69 | 2.09 | 0.60 | 1.32 | 1.36 | 3.08 | 0.38 | 4.71 | 7.36 | 6.45 | 9.13 | 1.77 |  |  |  |  |  |
|  |  |  |  |  |  |  |  |  |  |  |  |  |  |  |  |  |  |  |  |
|  |  | **Lymph Events** | **Lymph abs (cells/µl)** | **CD3 %L** | **TCRgd+ %L** | **Vg9+ %L** | **Vg9- %L** | **TCRgd- %L** | **CD8 %L** | **CD4+ CD8+ %L** | **CD4-CD8- %L** | **CD4 %L** | **CD4 conv %L** | **Treg %L** | **CD3+ CD56+ %L** | **CD3- %L** | **CD56+ %L** | **CD19+ %L** | **CD20+ %L** |
| Mastermix  1 day old | Donor 1 | 12664 | 2008.10 | 76.41 | 0.58 | 0.34 | 0.23 | 75.73 | 32.71 | 2.05 | 1.11 | 39.98 | 35.57 | 3.68 | 4.68 | 23.59 | 11.88 | 7.17 | 7.25 |
| Mastermix  1 day old | Donor 1 | 12602 | 1958.30 | 76.81 | 0.43 | 0.27 | 0.14 | 76.33 | 32.93 | 2.25 | 0.90 | 40.25 | 35.53 | 4.02 | 4.36 | 23.19 | 12.33 | 7.55 | 7.61 |
| Mastermix  1 month old | Donor 1 | 12925 | 1947.90 | 74.69 | 0.68 | 0.16 | 0.50 | 73.90 | 30.85 | 1.88 | 1.11 | 40.06 | 36.20 | 3.48 | 7.17 | 25.31 | 12.38 | 7.28 | 7.30 |
| Mastermix  1 month old | Donor 1 | 13154 | 1996.60 | 73.64 | 0.39 | 0.19 | 0.20 | 73.13 | 30.84 | 2.12 | 0.91 | 39.25 | 35.08 | 3.31 | 6.68 | 26.36 | 12.58 | 7.10 | 7.15 |
|  |  |  |  |  |  |  |  |  |  |  |  |  |  |  |  |  |  |  |  |
| Average deviation |  | 203.25 | 24.62 | 1.22 | 0.11 | 0.07 | 0.12 | 1.26 | 0.99 | 0.11 | 0.10 | 0.32 | 0.30 | 0.23 | 1.20 | 1.22 | 0.21 | 0.14 | 0.14 |
| Mean |  | 12836.25 | 1977.73 | 75.39 | 0.52 | 0.24 | 0.27 | 74.77 | 31.83 | 2.08 | 1.01 | 39.89 | 35.60 | 3.62 | 5.72 | 24.61 | 12.29 | 7.28 | 7.33 |
| Standard deviation |  | 219.88 | 25.23 | 1.29 | 0.12 | 0.07 | 0.14 | 1.30 | 0.99 | 0.13 | 0.10 | 0.38 | 0.40 | 0.26 | 1.22 | 1.29 | 0.26 | 0.17 | 0.17 |
| Variations-coefficient % |  | 1.71 | 1.28 | 1.71 | 22.39 | 29.32 | 51.62 | 1.74 | 3.11 | 6.43 | 10.18 | 0.95 | 1.12 | 7.29 | 21.32 | 5.22 | 2.08 | 2.35 | 2.34 |

**Supplementary Table 2A: Panel 1 mastermix stability.** BD multi-check control (upper table) and EDTA blood from a donor (lower table) was aliquoted and stained with antibody mastermix prepared 1 and 30 days prior to staining. BD multi-check control was used as reference. Donor samples (Row 9, Row 11) were performed as technical replicates. Abbreviation: L = Lymphocytes.

|  | **Material** | **Viable Lymph Events** | **CD3 % viaL** | **TCRgd % viaL** | **CD4 %viaL** | **CD8 %viaL** | **CD8+ TEMRA %viaL** | **CD8+naive %viaL** | **CD8+Eff/m %viaL** | **CD8+CM %viaL** | **CD4+CD8+ %viaL** | **CD4-CD8- %viaL** | **CD4+**  **conv %viaL** | **CD4+**  **conv**  **TEMRA %viaL** | **CD4+**  **conv**  **naive %viaL** | **CD4+**  **conv**  **Eff/m %viaL** | **CD4+**  **conv**  **CM %viaL** | **Treg %viaL** | **CD3+CD56+ %viaL** | **CD3- %viaL** | **CD56+ %viaL** | **CD19+ %viaL** |
| --- | --- | --- | --- | --- | --- | --- | --- | --- | --- | --- | --- | --- | --- | --- | --- | --- | --- | --- | --- | --- | --- | --- |
| Mastermix  1 day old | Donor 2 | 590283 | 67.69 | 0.48 | 32.54 | 30.55 | 10.66 | 6.97 | 10.20 | 2.73 | 2.16 | 1.95 | 29.21 | 3.84 | 7.61 | 10.43 | 7.33 | 3.07 | 8.40 | 32.31 | 16.82 | 7.82 |
| Mastermix  1 day old | Donor 2 | 579606 | 67.57 | 0.46 | 31.73 | 31.16 | 11.20 | 6.45 | 10.66 | 2.85 | 2.23 | 1.98 | 27.48 | 4.78 | 8.37 | 8.78 | 5.54 | 3.57 | 8.45 | 32.43 | 17.59 | 7.35 |
| Mastermix  1 month old | Donor 2 | 538151 | 69.90 | 0.49 | 35.46 | 30.09 | 10.94 | 7.40 | 8.61 | 3.13 | 2.28 | 1.57 | 31.66 | 4.50 | 9.30 | 9.32 | 8.55 | 3.36 | 8.04 | 30.10 | 16.16 | 7.36 |
| Mastermix  1 month old | Donor 2 | 529887 | 68.56 | 0.49 | 33.94 | 30.54 | 10.72 | 6.73 | 10.13 | 2.96 | 2.28 | 1.31 | 30.82 | 3.86 | 9.45 | 10.48 | 7.02 | 2.72 | 7.88 | 31.44 | 16.81 | 7.56 |
|  |  |  |  |  |  |  |  |  |  |  |  |  |  |  |  |  |  |  |  |  |  |  |
| Average deviation |  | 25462.75 | 0.80 | 0.01 | 1.28 | 0.29 | 0.19 | 0.30 | 0.65 | 0.13 | 0.04 | 0.26 | 1.45 | 0.40 | 0.69 | 0.70 | 0.83 | 0.29 | 0.23 | 0.80 | 0.37 | 0.17 |
| Mean |  | 559481.75 | 68.43 | 0.48 | 33.42 | 30.59 | 10.88 | 6.89 | 9.90 | 2.92 | 2.24 | 1.70 | 29.79 | 4.25 | 8.68 | 9.75 | 7.11 | 3.18 | 8.19 | 31.57 | 16.85 | 7.52 |
| Standard deviation |  | 25906.33 | 0.93 | 0.01 | 1.42 | 0.38 | 0.21 | 0.35 | 0.77 | 0.15 | 0.05 | 0.28 | 1.60 | 0.41 | 0.74 | 0.73 | 1.07 | 0.32 | 0.24 | 0.93 | 0.51 | 0.19 |
| Variations-coefficient % |  | 4.63 | 1.36 | 2.55 | 4.25 | 1.24 | 1.95 | 5.06 | 7.80 | 5.05 | 2.20 | 16.35 | 5.37 | 9.59 | 8.58 | 7.47 | 15.07 | 10.05 | 2.93 | 2.95 | 3.01 | 2.54 |

**Supplementary Table 2B: Panel 2 mastermix stability.** EDTA blood from a donor was aliquoted and stained with antibody mastermixes prepared 1 and 30 days prior to staining. Donor samples (Row 2, Row 4) were performed as technical replicates. Abbreviation: viaL= viable Lymphocytes.

| **Incubation time prior to staining** | **Lymph Events** | **Lymph abs (cells/µl)** | **Lymph abs**  **abs change** | **Lymph abs**  **rel change (%)** | **CD3 %L** | **CD3 %L**  **abs change** | **CD3 %L**  **rel change (%)** | **CD4 %L** | **CD4 %L**  **abs change** | **CD4 %L**  **rel change (%)** | **CD8 %L** | **CD8 %L**  **abs change** | **CD8 %L**  **rel change (%)** | **CD3- %L** | **CD3 %L**  **abs change** | **CD3- %L**  **rel change (%)** |
| --- | --- | --- | --- | --- | --- | --- | --- | --- | --- | --- | --- | --- | --- | --- | --- | --- |
| Donor 3 |  |  |  |  |  |  |  |  |  |  |  |  |  |  |  |  |
| 0h | 12428 | 1897.50 |  |  | 60.06 |  |  | 37.48 |  |  | 17.20 |  |  | 39.94 |  |  |
| 6h | 12584 | 1839.90 | -57.60 | -3.04 | 59.58 | -0.48 | -0.80 | 36.88 | -0.60 | -1.60 | 17.31 | 0.11 | 0.64 | 40.42 | 0.48 | 1.20 |
| 12h | 10953 | 1811.30 | -86.20 | -4.54 | 60.60 | 0.54 | 0.90 | 36.94 | -0.54 | -1.44 | 17.04 | -0.16 | -0.93 | 39.40 | -0.54 | -1.35 |
| 24h | 11160 | 1812.50 | -85.00 | -4.48 | 59.26 | -0.80 | -1.33 | 36.28 | -1.20 | -3.20 | 16.85 | -0.35 | -2.03 | 40.74 | 0.80 | 2.00 |
| 48h | 12660 | 2002.90 | 105.40 | 5.55 | 56.53 | -3.53 | -5.88 | 35.69 | -1.79 | -4.78 | 15.69 | -1.51 | -8.78 | 43.47 | 3.53 | 8.84 |
|  |  |  |  |  |  |  |  |  |  |  |  |  |  |  |  |  |
| Donor 4 |  |  |  |  |  |  |  |  |  |  |  |  |  |  |  |  |
| 0h | 12254 | 1736.10 |  |  | 67.03 |  |  | 43.38 |  |  | 8.92 |  |  | 32.97 |  |  |
| 6h | 11963 | 1714.30 | -21.80 | -1.26 | 67.10 | 0.07 | 0.10 | 44.75 | 1.37 | 3.16 | 8.11 | -0.81 | -9.08 | 32.90 | -0.07 | -0.21 |
| 12h | 11254 | 1733.40 | -2.70 | -0.16 | 71.59 | 4.56 | 6.80 | 47.17 | 3.79 | 8.74 | 8.20 | -0.72 | -8.07 | 29.71 | -3.26 | -9.89 |
| 24h | 11168 | 1812.60 | 76.50 | 4.41 | 69.33 | 2.30 | 3.43 | 45.49 | 2.11 | 4.86 | 8.45 | -0.47 | -5.27 | 30.67 | -2.30 | -6.98 |
| 48h | 10749 | 1693.50 | -42.60 | -2.45 | 68.83 | 1.80 | 2.69 | 46.12 | 2.74 | 6.32 | 8.21 | -0.71 | -7.96 | 31.17 | -1.80 | -5.46 |
|  |  |  |  |  |  |  |  |  |  |  |  |  |  |  |  |  |
| Donor 5 |  |  |  |  |  |  |  |  |  |  |  |  |  |  |  |  |
| 0h | 22540 | 3200.90 |  |  | 85.00 |  |  | 12.02 |  |  | 69.33 |  |  | 15.00 |  |  |
| 6h | 22337 | 3396.70 | 195.80 | 6.12 | 85.38 | 0.38 | 0.45 | 12.03 | 0.01 | 0.08 | 69.89 | 0.56 | 0.81 | 14.62 | -0.38 | -2.53 |
| 12h | 21907 | 3389.20 | 188.30 | 5.88 | 85.00 | 0.00 | 0.00 | 12.24 | 0.22 | 1.83 | 69.32 | -0.01 | -0.01 | 15.00 | 0.00 | 0.00 |
| 24h | 20118 | 3474.40 | 273.50 | 8.54 | 84.07 | -0.93 | -1.09 | 11.89 | -0.13 | -1.08 | 68.39 | -0.94 | -1.36 | 15.93 | 0.93 | 6.20 |
| 48h | 20171 | 3013.00 | -187.90 | -5.87 | 84.51 | -0.49 | -0.58 | 12.00 | -0.02 | -0.17 | 69.03 | -0.30 | -0.43 | 15.49 | 0.49 | 3.27 |

**Supplementary Table 3A: Sample stability panel 1.** Donor EDTA blood was incubated at room temperature for the listed amount of time prior to staining. Absolute and relative change (%) compare values to 0h incubation time prior to staining. Abbreviation: L = Lymphocytes.

| **Incubation time prior to staining** | **viable Lymph Events** | **CD3 % viaL** | **CD3 % viaL**  **abs change** | **CD3 % viaL**  **rel change (%)** | **CD4 % viaL** | **CD4 % viaL**  **abs change** | **CD4 % viaL**  **rel change (%)** | **CD8 % viaL** | **CD8 % viaL**  **abs change** | **CD8 % viaL**  **rel change (%)** | **CD3- % viaL** | **CD3- % vial**  **abs change** | **CD3- % viaL**  **rel change (%)** |
| --- | --- | --- | --- | --- | --- | --- | --- | --- | --- | --- | --- | --- | --- |
| Donor 6 |  |  |  |  |  |  |  |  |  |  |  |  |  |
| 0h | 50000 | 46.70 |  |  | 28.11 |  |  | 14.22 |  |  | 53.3 |  |  |
| 6h | 49825 | 44.82 | -1.88 | -4.03 | 25.57 | -2.54 | -9.04 | 14.11 | -0.11 | -0.77 | 55.18 | 1.88 | 3.53 |
| 12h | 49357 | 45.22 | -1.48 | -3.17 | 26.50 | -1.61 | -5.73 | 14.00 | -0.22 | -1.55 | 54.78 | 1.48 | 2.78 |
| 24h | 49785 | 45.60 | -1.10 | -2.36 | 28.04 | -0.07 | -0.25 | 13.04 | -1.18 | -8.30 | 54.40 | 1.10 | 2.06 |
| **48h** | 49613 | 55.92 | 9.22 | **19.74** | 36.01 | 7.90 | **28.10** | 15.62 | 1.40 | 9.85 | 44.08 | -9.22 | **-17.30** |
|  |  |  |  |  |  |  |  |  |  |  |  |  |  |
| Donor 7 |  |  |  |  |  |  |  |  |  |  |  |  |  |
| 0h | 45202 | 65.06 |  |  | 42.30 |  |  | 8.16 |  |  | 34.94 |  |  |
| 6h | 43592 | 62.75 | -2.31 | -3.55 | 39.01 | -3.29 | -7.78 | 7.95 | -0.21 | -2.57 | 37.25 | 2.31 | 6.61 |
| 12h | 46589 | 65.76 | 0.70 | 1.08 | 42.28 | -0.02 | -0.05 | 8.11 | -0.05 | -0.61 | 34.24 | -0.70 | -2.00 |
| 24h | 38256 | 65.29 | 0.23 | 0.35 | 41.44 | -0.86 | -2.03 | 7.74 | -0.42 | -5.15 | 34.71 | -0.23 | -0.66 |
| **48h** | 22247 | 79.60 | 14.54 | **22.35** | 62.41 | 20.11 | **47.54** | 9.25 | 1.09 | **13.36** | 20.40 | -14.54 | **-41.61** |
|  |  |  |  |  |  |  |  |  |  |  |  |  |  |
| Donor 8 |  |  |  |  |  |  |  |  |  |  |  |  |  |
| 0h | 50001 | 81.67 |  |  | 11.20 |  |  | 67.56 |  |  | 18.33 |  |  |
| 6h | 49825 | 83.02 | 1.35 | 1.65 | 12.11 | 0.91 | 8.13 | 68.41 | 0.85 | 1.26 | 16.98 | -1.35 | -7.36 |
| 12h | 49699 | 83.30 | 1.63 | 2.00 | 11.60 | 0.40 | 3.57 | 68.29 | 0.73 | 1.08 | 16.70 | -1.63 | -8.89 |
| 24h | 49324 | 81.80 | 0.13 | 0.16 | 11.58 | 0.38 | 3.39 | 71.25 | 3.69 | 5.46 | 18.20 | -0.13 | -0.71 |
| **48h** | 51079 | 90.79 | 9.12 | **11.17** | 13.42 | 2.22 | **19.82** | 74.19 | 6.63 | 9.81 | 9.21 | -9.12 | **-49.75** |

**Supplementary Table 3B: Sample stability panel 2**. Donor EDTA blood was incubated at room temperature for the listed amount of time prior to staining. Relative change >10% marked in red. Absolute and relative change (%) compare values to 0h incubation time prior to staining. Abbreviation: viaL= viable Lymphocytes.

| **Dilution** | **Lymph Events** | **Lymph abs (cells/µl)** | **CD3 %L** | **CD3 %L**  **abs change** | **CD3 %L**  **rel change (%)** | **CD4 %L** | **CD4 %L**  **abs change** | **CD4 %L**  **rel change (%)** | **CD8 %L** | **CD8 %L**  **abs change** | **CD8 %L**  **rel change (%)** | **CD3- %L** | **CD3 %L**  **abs change** | **CD3- %L**  **rel change (%)** |
| --- | --- | --- | --- | --- | --- | --- | --- | --- | --- | --- | --- | --- | --- | --- |
| Donor 9 |  |  |  |  |  |  |  |  |  |  |  |  |  |  |
| 1:1 | 12428 | 1897.50 | 53.06 |  |  | 33.48 |  |  | 16.20 |  |  | 46.94 |  |  |
| 1:2 | 8258 | 1064.30 | 52.94 | -0.12 | -0.23 | 32.61 | -0.87 | -2.60 | 15.40 | -0.80 | -4.94 | 47.06 | 0.12 | 0.26 |
| 1:5 | 4090 | 495.60 | 52.17 | -0.89 | -1.68 | 32.24 | -1.24 | -3.70 | 15.79 | -0.41 | -2.53 | 47.83 | 0.89 | 1.90 |
| 1:10 | 2184 | 247.00 | 52.68 | -0.38 | -0.72 | 32.82 | -0.66 | -1.97 | 15.54 | -0.66 | -4.07 | 47.32 | 0.38 | 0.81 |
| 1:20 | 1516 | 177.80 | 48.48 | -4.58 | -8.63 | 31.53 | -1.95 | -5.82 | 15.45 | -0.75 | -4.63 | 51.52 | 4.58 | 9.76 |
| **1:50** | 602 | 69.60 | 52.99 | -0.07 | -0.13 | 31.40 | -2.08 | -6.21 | 13.65 | -2.55 | **-15.74** | 47.01 | 0.07 | 0.15 |
| **1:100** | 150 | 18.30 | 52.00 | -1.06 | -2.00 | 26.67 | -6.81 | **-20.34** | 18.00 | 1.80 | **11.11** | 48.00 | 1.06 | 2.26 |
| **Empty** | 0 | 0.00 | 0.00 | -53.06 | **-100.00** | 0.00 | -33.48 | **-100.00** | 0.00 | -16.20 | **-100.00** | 0.00 | -46.94 | **-100.00** |
|  |  |  |  |  |  |  |  |  |  |  |  |  |  |  |
| Donor 10 |  |  |  |  |  |  |  |  |  |  |  |  |  |  |
| 1:1 | 12254 | 1736.10 | 67.03 |  |  | 43.38 |  |  | 8.92 |  |  | 32.97 |  |  |
| 1:2 | 6976 | 863.80 | 66.46 | -0.57 | -0.85 | 44.02 | 0.64 | 1.48 | 8.16 | -0.76 | -8.52 | 33.54 | 0.57 | 1.73 |
| 1:5 | 3183 | 367.60 | 67.47 | 0.44 | 0.66 | 42.79 | -0.59 | -1.36 | 8.17 | -0.75 | -8.41 | 32.53 | -0.44 | -1.33 |
| 1:10 | **1776** | 198.00 | 68.02 | 0.99 | 1.48 | 46.62 | 3.24 | 7.47 | 8.12 | -0.80 | -8.97 | 31.98 | -0.99 | -3.00 |
| **1:20** | 981 | 116.50 | 68.30 | 1.27 | 1.89 | 44.75 | 1.37 | 3.16 | 7.34 | -1.58 | **-17.71** | 31.70 | -1.27 | -3.85 |
| **1:50** | 320 | 36.20 | 68.44 | 1.41 | 2.10 | 43.75 | 0.37 | 0.85 | 5.62 | -3.30 | **-37.00** | 31.56 | -1.41 | -4.28 |
| **1:100** | 105 | 11.80 | 64.76 | -2.27 | -3.39 | 43.81 | 0.43 | 0.99 | 3.81 | -5.11 | **-57.29** | 35.24 | 2.27 | 6.89 |
| **Empty** | 0 | 0.00 | 0.00 | -67.03 | **-100.00** | 0.00 | -43.38 | **-100.00** | 0.00 | -8.92 | **-100.00** | 0.00 | -32.97 | **-100.00** |
|  |  |  |  |  |  |  |  |  |  |  |  |  |  |  |
| Donor 11 |  |  |  |  |  |  |  |  |  |  |  |  |  |  |
| 1:1 | 5902 | 846.70 | 91.51 |  |  | 22.18 |  |  | 57.23 |  |  | 8.49 |  |  |
| 1:2 | 3485 | 437.90 | 91.13 | -0.38 | -0.42 | 21.84 | -0.34 | -1.53 | 56.73 | -0.50 | -0.87 | 8.87 | 0.38 | 4.48 |
| 1:5 | 1140 | 168.40 | 91.67 | 0.16 | 0.17 | 22.29 | 0.11 | 0.50 | 56.94 | -0.29 | -0.51 | 8.33 | -0.16 | -1.88 |
| 1:10 | 880 | 100.50 | 91.14 | -0.37 | -0.40 | 22.50 | 0.32 | 1.44 | 54.66 | -2.57 | -4.49 | 8.90 | 0.41 | 4.83 |
| **1:20** | 469 | 55.10 | 90.19 | -1.32 | -1.44 | 24.52 | 2.34 | **10.55** | 51.39 | -5.84 | **-10.20** | 9.81 | 1.32 | **15.55** |
| **1:50** | 219 | 25.40 | 87.21 | -4.30 | -4.70 | 20.09 | -2.09 | -9.42 | 49.32 | -7.91 | **-13.82** | 8.68 | 0.19 | 2.24 |
| **1:100** | 72 | 8.50 | 83.33 | -8.18 | -8.94 | 11.11 | -11.07 | **-49.91** | 55.56 | -1.67 | -2.92 | 16.67 | 8.18 | **96.35** |
| **Empty** | 0 | 0.00 | 0.00 | -91.51 | **-100.00** | 0.00 | -22.18 | **-100.00** | 0.00 | -57.23 | **-100.00** | 0.00 | -8.49 | **-100.00** |

**Supplementary Table 4A: Panel 1 linearity and diagnostic threshold.** Samples were diluted in indicated ratios and stained. Relative change >10% is marked in red. Minimal diagnostic threshold (marked in green) was identified by the highest required lymphocyte events at the last stable dilution step (1:10, donor 10 = 1776 lymphocyte events). Absolute and relative change (%) compare values to the 1:1 dilution step. Abbreviation: L = Lymphocytes.

| **Dilution** | **viable Lymph Events** | **CD3 % viaL** | **CD3 % viaL**  **abs change** | **CD3 % viaL**  **rel change (%)** | **CD4 % viaL** | **CD4 % viaL**  **abs change** | **CD4 % viaL**  **rel change (%)** | **CD8 % viaL** | **CD8 % viaL**  **abs change** | **CD8 % viaL**  **rel change (%)** | **CD3- % viaL** | **CD3- % vial**  **abs change** | **CD3- % viaL**  **rel change (%)** |
| --- | --- | --- | --- | --- | --- | --- | --- | --- | --- | --- | --- | --- | --- |
| Donor 12 |  |  |  |  |  |  |  |  |  |  |  |  |  |
| 1:1 | 50039 | 47.96 |  |  | 29.64 |  |  | 13.99 |  |  | 52.04 |  |  |
| 1:2 | 28383 | 47.15 | -0.81 | -1.69 | 27.79 | -1.85 | -6.24 | 14.69 | 0.70 | 5.00 | 52.85 | 0.81 |  |
| 1:5 | 12841 | 46.79 | -1.17 | -2.44 | 26.94 | -2.70 | -9.11 | 14.97 | 0.98 | 7.01 | 53.21 | 1.17 | 2.25 |
| 1:10 | **5536** | 48.66 | 0.70 | 1.46 | 26.73 | -2.91 | -9.82 | 14.83 | 0.84 | 6.00 | 51.34 | -0.70 | -1.35 |
| **1:20** | 2620 | 46.85 | -1.11 | -2.31 | 25.80 | -3.84 | **-12.96** | 14.27 | 0.28 | 2.00 | 53.15 | 1.11 | 2.13 |
| **1:50** | 222 | 54.50 | 6.54 | **13.64** | 34.23 | 4.59 | **15.49** | 15.32 | 1.33 | 9.51 | 45.50 | -6.54 | **-12.57** |
| **1:100** | 10 | 30.00 | -17.96 | **-37.45** | 10.00 | -19.64 | **-66.26** | 0.00 | -13.99 | **-100.00** | 70.00 | 17.96 | **34.51** |
| **Empty** | 0 | 0.00 | -47.96 | **-100.00** | 0.00 | -29.64 | **-100.00** | 0.00 | -13.99 | **-100.00** | 0.00 | -52.04 | **-100.00** |
|  |  |  |  |  |  |  |  |  |  |  |  |  |  |
| Donor 13 |  |  |  |  |  |  |  |  |  |  |  |  |  |
| 1:1 | 45202 | 65.06 |  |  | 42.30 |  |  | 8.16 |  |  | 34.94 |  |  |
| 1:2 | 25953 | 66.31 | 1.25 | 1.92 | 42.49 | 0.19 | 0.45 | 8.35 | 0.19 | 2.33 | 33.69 | -1.25 | -3.58 |
| 1:5 | 10313 | 66.15 | 1.09 | 1.68 | 42.02 | -0.28 | -0.66 | 8.48 | 0.32 | 3.92 | 33.85 | -1.09 | -3.12 |
| 1:10 | 4711 | 64.94 | -0.12 | -0.18 | 39.33 | -2.97 | -7.02 | 8.49 | 0.33 | 4.04 | 35.06 | 0.12 | 0.34 |
| **1:20** | 1817 | 68.24 | 3.18 | 4.89 | 40.12 | -2.18 | -5.15 | 9.69 | 1.53 | **18.75** | 31.76 | -3.18 | -9.10 |
| **1:50** | 234 | 59.83 | -5.23 | -8.04 | 33.33 | -8.97 | **-21.21** | 7.26 | -0.90 | **-11.03** | 40.17 | 5.23 | **14.97** |
| **1:100** | 5 | 0.00 | -65.06 | **-100.00** | 0.00 | -42.30 | **-100.00** | 0.00 | -8.16 | **-100.00** | 0.00 | -34.94 | **-100.00** |
| **Empty** | 0 | 0.00 | -65.06 | **-100.00** | 0.00 | -42.30 | **-100.00** | 0.00 | -8.16 | **-100.00** | 0.00 | -34.94 | **-100.00** |
|  |  |  |  |  |  |  |  |  |  |  |  |  |  |
| Donor 14 |  |  |  |  |  |  |  |  |  |  |  |  |  |
| 1:1 | 23291 | 89.00 |  |  | 22.15 |  |  | 52.13 |  |  | 11.00 |  |  |
| 1:2 | 12727 | 88.69 | -0.31 | -0.35 | 21.41 | -0.74 | -3.34 | 50.60 | -1.53 | -2.93 | 11.31 | 0.31 | 2.82 |
| 1:5 | 3330 | 88.53 | -0.47 | -0.53 | 22.75 | 0.60 | 2.71 | 52.69 | 0.56 | 1.07 | 11.47 | 0.47 | 4.27 |
| 1:10 | 2005 | 88.18 | -0.82 | -0.92 | 21.10 | -1.05 | -4.74 | 52.77 | 0.64 | 1.23 | 11.82 | 0.82 | 7.45 |
| 1:20 | 1457 | 88.47 | -0.53 | -0.60 | 21.41 | -0.74 | -3.34 | 51.48 | -0.65 | -1.25 | 11.53 | 0.53 | 4.82 |
| 1:50 | 327 | 88.38 | -0.62 | -0.70 | 20.18 | -1.97 | -8.89 | 55.66 | 3.53 | 6.77 | 11.62 | 0.62 | 5.64 |
| **1:100** | 95 | 84.21 | -4.79 | -5.38 | 16.84 | -5.31 | **-23.97** | 56.84 | 4.71 | 9.04 | 15.79 | 4.79 | **43.55** |
| **Empty** | 0 | 0.00 | -89.00 | **-100.00** | 0.00 | -22.15 | **-100.00** | 0.00 | -52.13 | **-100.00** | 0.00 | -11.00 | **-100.00** |

**Supplementary Table 4B: Panel 2 linearity and diagnostic threshold.** Samples were diluted in indicated ratios and stained. Relative change >10% are marked in red. Minimal diagnostic threshold (marked in green) was identified by the highest required lymphocyte events at the last stable dilution step (1:10, donor 10 = 5536 lymphocyte events). Absolute and relative change (%) compare values to 0h incubation time prior to staining. Abbreviation: viaL= viable Lymphocytes.

|  | **Lot number** | **Lymph/µl** | **CD3%L** | **CD3/µl** | **CD4%L** | **CD4/µl** | **CD8%L** | **CD8/µl** | **CD56%L** | **CD56/µl** | **CD19%L** | **CD19/µl** | **CD20%L** |
| --- | --- | --- | --- | --- | --- | --- | --- | --- | --- | --- | --- | --- | --- |
| BD multi-check control (Reference) | BM0419N | 1680 | 74.2 (64.2-84.2) | 1246.6 (997.2-1495.9) | 47.7 (41.2-54.2) | 801.4 (641.1-961.6) | 24.4 (17.4-31.4) | 409.9 (295.6-524.3) | 11.7 (4.7-18.7) | 196.6 (104.2-288.9) | 12.6 (8.6-16.6) | 211.7 (146.5-276.9) | 13.3 (8.3-18.3) |
|  |  |  |  |  |  |  |  |  |  |  |  |  |  |
| Replicate 1 | BM0419N | 1695 | 75.09 | 1272.60 | 49.76 | 843.30 | 20.43 | 346.20 | 8.53 | 144.60 | 15.49 | 262.60 | 13.06 |
| Replicate 2 | BM0419N | 1764 | 74.36 | 1311.40 | 47.87 | 844.20 | 20.20 | 356.20 | 9.39 | 165.60 | 15.35 | 270.80 | 13.22 |
| Replicate 3 | BM0419N | 1768 | 75.82 | 1340.50 | 51.09 | 903.20 | 21.66 | 383.00 | 8.71 | 154.00 | 14.95 | 264.40 | 12.59 |
| Replicate 4 | BM0419N | 1784 | 76.17 | 1358.50 | 49.65 | 885.60 | 20.47 | 365.20 | 8.79 | 156.70 | 15.07 | 268.80 | 12.66 |
| Replicate 5 | BM0419N | 1774 | 75.65 | 1341.90 | 47.60 | 844.50 | 20.42 | 362.20 | 8.31 | 147.40 | 15.17 | 269.20 | 12.44 |
| Replicate 6 | BM0419N | 1708 | 75.16 | 1283.40 | 49.22 | 840.50 | 19.87 | 339.30 | 9.09 | 155.20 | 15.41 | 263.20 | 12.69 |
| Replicate 7 | BM0419N | 1771 | 75.12 | 1330.00 | 47.24 | 836.40 | 20.48 | 362.70 | 9.38 | 166.10 | 15.27 | 270.40 | 13.12 |
| Replicate 8 | BM0419N | 1690 | 75.35 | 1273.40 | 49.47 | 835.90 | 21.00 | 354.90 | 8.94 | 151.10 | 15.12 | 255.50 | 12.53 |
| Replicate 9 | BM0419N | 1715 | 75.61 | 1297.00 | 48.36 | 829.60 | 19.80 | 339.70 | 8.94 | 153.40 | 15.07 | 258.60 | 12.59 |
| Replicate 10 | BM0419N | 1684 | 74.91 | 1261.70 | 48.21 | 812.00 | 20.94 | 352.80 | 8.94 | 150.50 | 14.93 | 251.50 | 12.85 |
|  |  |  |  |  |  |  |  |  |  |  |  |  |  |
| Average deviation |  | 36.78 | 0.40 | 29.42 | 0.99 | 18.75 | 0.40 | 9.64 | 0.25 | 5.15 | 0.16 | 5.22 | 0.23 |
| Mean |  | 1735.20 | 75.32 | 1307.04 | 48.85 | 847.52 | 20.53 | 356.22 | 8.90 | 154.46 | 15.18 | 263.50 | 12.78 |
| Standard deviation |  | 37.95 | 0.49 | 32.50 | 1.13 | 25.45 | 0.53 | 12.44 | 0.32 | 6.64 | 0.18 | 6.29 | 0.26 |
| Variationscoefficient % |  | 2.19 | 0.65 | 2.49 | 2.31 | 3.00 | 2.56 | 3.49 | 3.63 | 4.30 | 1.20 | 2.39 | 2.02 |

**Supplementary Table 5A: Panel 1 precision.** BD multi-check control was aliquoted (10 replicates) and stained. Abbreviation: L = Lymphocytes.

|  | **Viable Lymph Events** | **CD3 % viaL** | **TCRgd % viaL** | **CD4 %viaL** | **CD8 %viaL** | **CD8+ TEMRA %viaL** | **CD8+**  **naive %viaL** | **CD8+**  **Eff/m %viaL** | **CD8+CM %viaL** | **CD4+CD8+ %viaL** | **CD4-CD8- %viaL** | **CD4+**  **conv %viaL** | **CD4+**  **conv**  **TEMRA %viaL** | **CD4+**  **conv**  **naive %viaL** | **CD4+**  **conv**  **Eff/m %viaL** | **CD4+**  **conv**  **CM %viaL** | **Treg %viaL** | **CD3+CD56+ %viaL** | **CD3- %viaL** | **CD56+ %viaL** | **CD19+ %viaL** |
| --- | --- | --- | --- | --- | --- | --- | --- | --- | --- | --- | --- | --- | --- | --- | --- | --- | --- | --- | --- | --- | --- |
| Replicate 1 | 45288 | 66.24 | 13.95 | 43.48 | 8.15 | 2.09 | 1.38 | 3.44 | 1.23 | 0.18 | 0.48 | 40.05 | 0.29 | 27.19 | 3.79 | 8.79 | 2.86 | 10.69 | 33.76 | 14.64 | 14.91 |
| Replicate 2 | 49726 | 65.55 | 14.15 | 42.77 | 7.73 | 1.95 | 1.27 | 3.30 | 1.21 | 0.26 | 0.63 | 39.49 | 0.46 | 26.55 | 3.77 | 8.72 | 2.91 | 10.76 | 34.45 | 14.57 | 15.35 |
| Replicate 3 | 38048 | 64.95 | 14.36 | 41.87 | 7.86 | 1.97 | 1.32 | 3.21 | 1.36 | 0.18 | 0.68 | 38.44 | 0.44 | 24.17 | 3.58 | 10.24 | 2.86 | 11.10 | 35.05 | 15.03 | 15.40 |
| Replicate 4 | 44701 | 64.98 | 14.25 | 41.97 | 7.99 | 2.02 | 1.36 | 3.39 | 1.22 | 0.23 | 0.55 | 38.67 | 0.49 | 26.08 | 3.51 | 8.60 | 2.79 | 10.93 | 35.02 | 14.83 | 15.65 |
| Replicate 5 | 49722 | 66.53 | 13.94 | 43.75 | 8.14 | 2.03 | 1.37 | 3.50 | 1.23 | 0.19 | 0.52 | 40.39 | 0.58 | 26.84 | 3.94 | 9.04 | 2.81 | 10.57 | 33.47 | 14.33 | 14.99 |
| Replicate 6 | 49644 | 66.03 | 14.14 | 42.97 | 8.20 | 2.12 | 1.33 | 3.47 | 1.28 | 0.26 | 0.46 | 39.60 | 0.55 | 26.97 | 3.50 | 8.58 | 2.79 | 10.86 | 33.97 | 14.58 | 15.16 |
| Replicate 7 | 49517 | 66.33 | 13.96 | 43.47 | 8.18 | 2.01 | 1.41 | 3.44 | 1.33 | 0.18 | 0.53 | 39.98 | 0.45 | 27.19 | 3.67 | 8.69 | 2.91 | 10.49 | 33.67 | 14.24 | 15.19 |
| Replicate 8 | 49573 | 65.73 | 14.34 | 42.80 | 7.88 | 1.94 | 1.40 | 3.28 | 1.26 | 0.23 | 0.48 | 39.40 | 0.60 | 26.56 | 3.53 | 8.71 | 2.82 | 10.75 | 34.27 | 14.49 | 15.42 |
| Replicate 9 | 49474 | 66.17 | 13.85 | 43.61 | 8.00 | 2.03 | 1.38 | 3.31 | 1.28 | 0.24 | 0.47 | 40.28 | 0.42 | 26.99 | 3.73 | 9.14 | 2.75 | 10.53 | 33.83 | 14.47 | 15.19 |
| Replicate 10 | 49714 | 66.22 | 14.08 | 43.53 | 7.93 | 1.72 | 1.80 | 2.76 | 1.64 | 0.22 | 0.46 | 39.96 | 0.77 | 26.91 | 3.45 | 8.83 | 2.86 | 10.60 | 33.78 | 14.39 | 15.13 |
|  |  |  |  |  |  |  |  |  |  |  |  |  |  |  |  |  |  |  |  |  |  |
| Average deviation | 2917.02 | 0.46 | 0.15 | 0.55 | 0.13 | 0.07 | 0.08 | 0.14 | 0.08 | 0.03 | 0.06 | 0.51 | 0.10 | 0.57 | 0.13 | 0.32 | 0.04 | 0.15 | 0.46 | 0.17 | 0.17 |
| Mean | 47540.70 | 65.87 | 14.10 | 43.02 | 8.01 | 1.99 | 1.40 | 3.31 | 1.30 | 0.22 | 0.53 | 39.63 | 0.51 | 26.55 | 3.65 | 8.93 | 2.84 | 10.73 | 34.13 | 14.56 | 15.24 |
| Standard deviation | 3656.56 | 0.53 | 0.17 | 0.64 | 0.15 | 0.10 | 0.14 | 0.20 | 0.12 | 0.03 | 0.07 | 0.62 | 0.12 | 0.85 | 0.15 | 0.47 | 0.05 | 0.18 | 0.53 | 0.22 | 0.21 |
| Variations-coefficient % | 7.69 | 0.80 | 1.19 | 1.49 | 1.88 | 5.25 | 9.87 | 6.16 | 9.29 | 14.14 | 13.61 | 1.56 | 24.24 | 3.22 | 4.12 | 5.23 | 1.77 | 1.71 | 1.55 | 1.53 | 1.37 |

**Supplementary Table 5B: Panel 2 precision.** Donor EDTA blood was aliquoted (10 replicates) and stained. Abbreviation: viaL= viable Lymphocytes.

| **BD^TM^ FACSCanto II vs BD^TM^ FACSLyric** | **Lymph/µl** | **CD3%L** | **CD3/µl** | **CD4%L** | **CD4/µl** | **CD8%L** | **CD8/µl** | **CD19%L** | **CD19/µl** |
| --- | --- | --- | --- | --- | --- | --- | --- | --- | --- |
| Donor 15-Canto | 927 | 88.40 | 819.73 | 26.63 | 246.95 | 61.35 | 568.90 | 0.00 | 0.00 |
| Donor 15-Lyric | 881 | 89.74 | 790.80 | 26.54 | 233.80 | 62.05 | 546.70 | 0.00 | 0.00 |
|  |  |  |  |  |  |  |  |  |  |
| Donor 16-Canto | 538 | 84.01 | 451.66 | 24.83 | 133.50 | 59.91 | 322.10 | 0.00 | 0.00 |
| Donor 16-Lyric | 546 | 82.61 | 450.70 | 22.63 | 123.50 | 54.05 | 294.90 | 0.00 | 0.00 |
|  |  |  |  |  |  |  |  |  |  |
| Donor 17-Canto | 382 | 95.38 | 364.70 | 17.81 | 68.10 | 75.20 | 287.50 | 0.04 | 0.16 |
| Donor 17-Lyric | 388 | 96.01 | 372.80 | 17.04 | 66.20 | 72.29 | 280.70 | 0.00 | 0.00 |
|  |  |  |  |  |  |  |  |  |  |
| Donor 18-Canto | 295 | 85.71 | 253.00 | 76.08 | 224.60 | 9.37 | 27.70 | 0.04 | 0.13 |
| Donor 18-Lyric | 285 | 93.41 | 266.60 | 82.49 | 235.40 | 7.34 | 21.00 | 0.00 | 0.00 |

**Supplementary Table 6A: Comparison between BD^TM^ FACSCanto II vs BD^TM^ FACSLyric.** CD3, CD4, CD8 and CD19 measured with formerly used panel within the department (6-color TBNK on BD^TM^ FACS CANTO II) and the new panel 1 measured via BD^TM^ FACSLyric. All donors were previously treated with rituximab. Abbreviation: L = Lymphocytes.

| **Sysmex XN-9100^TM^ vs BD^TM^ FACSLyric** | **Lymph/µl** |
| --- | --- |
| Donor 19-Sysmex | 3000 |
| Donor 19-Lyric | 3201 |
|  |  |
| Donor 20-Sysmex | 2000 |
| Donor 20-Lyric | 1906 |
|  |  |
| Donor 21-Sysmex | 1700 |
| Donor 21-Lyric | 1635 |
|  |  |
| Donor 22-Sysmex | 1050 |
| Donor 22-Lyric | 979 |

**Supplementary Table 6B: Comparison between Sysmex XN-9100^TM^ vs BD^TM^ FACSLyric**. Absolute lymphocyte count measured with BD^TM^ FACSLyric (panel 1) compared to Sysmex XN-9100.

|  | | Males | | | | | | | Females | | | | | | |  |
| --- | --- | --- | --- | --- | --- | --- | --- | --- | --- | --- | --- | --- | --- | --- | --- | --- |
|  | 18-40 years old | | | |  | 41-69 years old | | | 18-40 years old | | |  | 41-69 years old | | | |
| *CD4_conv_* | 33.1 | |  | (17.6-48.9) |  | 31.1 |  | (13.4-46.6) | 39.3 |  | (23.6-54.8) |  | 37.4 |  | (18.7-57.5) | |
| *T_regs_* | 3.9 | |  | (1.2-6.9) |  | 4.7 |  | (2.2-8.3) | 3.3 |  | (1.4-7.8) |  | 4.2 |  | (1.4-8) | |
| *CD8^+^CD4^+^* | 0.3 | |  | (0.1-2.8) |  | 0.3 |  | (0.1-2.1) | 0.3 |  | (0.1-1.6) |  | 0.4 |  | (0.1-1.7) | |
| *CD8^+^* | 21.0 | |  | (11.1-34.5) |  | 16.4 |  | (7.2-36.1) | 19.6 |  | (10.5-38.8) |  | 16.8 |  | (10.3-34) | |
| *γδ T cells* | 2.9 | |  | (0.7-26.9) |  | 2.0 |  | (0.5-9.3) | 2.8 |  | (0.8-10.9) |  | 1.9 |  | (0.3-8.5) | |
| *Vγ9^+^* | 78.0 | |  | (7.6-98) |  | 69.2 |  | (19.1-95.8) | 73.8 |  | (33.3-95.1) |  | 68.3 |  | (12.3-94.1) | |
| *Vγ9^-^* | 21.0 | |  | (1.8-90.4) |  | 28.9 |  | (2.8-80.6) | 23.7 |  | (4.2-66.6) |  | 30.7 |  | (4.5-87.1) | |
| *B cells* | 12.1 | |  | (3.1-26.7) |  | 13.5 |  | (6.1-31.6) | 11.8 |  | (4.5-22.3) |  | 12.9 |  | (5.4-28.7) | |
| *CD20^+^* | 96.7 | |  | (89.8-99) |  | 96.7 |  | (87.0-98.8) | 97.9 |  | (90.0-99.4) |  | 97.6 |  | (91.3-99.3) | |
| *CD20^-^* | 3.0 | |  | (0.9-9.5) |  | 2.9 |  | (1.0-12.2) | 1.8 |  | (0.6-9.6) |  | 2.2 |  | (0.5-7.4) | |
| *NK cells* | 8.9 | |  | (2.3-22.4) |  | 12.0 |  | (2.2-41.5) | 9.1 |  | (1.9-26.9) |  | 10.1 |  | (3.6-29) | |
| *NKT cells* | 1.7 | |  | (0.3-10.1) |  | 1.3 |  | (0.1-15.4) | 1.4 |  | (0.2-7.5) |  | 1.4 |  | (0.2-7.3) | |
|  |  | |  |  |  |  |  |  |  |  |  |  |  |  |  | |
| *CD4conv* |  | |  |  |  |  |  |  |  |  |  |  |  |  |  | |
| *naive* | 54.0 | |  | (25.1-76.9) |  | 43.6 |  | (13.3-78.7) | 53.2 |  | (27.1-80.4) |  | 50.0 |  | (22.9-77.3) | |
| *cm* | 29.8 | |  | (0.3-51.1) |  | 33.0 |  | (14.8-51.3) | 28.6 |  | (0.9-50.3) |  | 32.5 |  | (12.9-52.8) | |
| *em* | 13.8 | |  | (0.2-28) |  | 20.1 |  | (6.8-43) | 14.0 |  | (0.5-24.4) |  | 15.8 |  | (6.2-30) | |
| *temra* | 1.0 | |  | (0.2-23.2) |  | 1.2 |  | (0.4-7.2) | 0.8 |  | (0.2-17.6) |  | 1.2 |  | (0.3-4.5) | |
|  |  | |  |  |  |  |  |  |  |  |  |  |  |  |  | |
| *Tregs* |  | |  |  |  |  |  |  |  |  |  |  |  |  |  | |
| *naive* | 43.7 | |  | (26.3-90.4) |  | 25.7 |  | (7.1-48.9) | 42.3 |  | (22.6-88.4) |  | 28.3 |  | (10.8-56.4) | |
| *cm* | 49.4 | |  | (1.3-66) |  | 65.9 |  | (43.2-83.6) | 51.6 |  | (3.2-68.7) |  | 63.9 |  | (38.8-80.7) | |
| *em* | 6.4 | |  | (0.5-13.5) |  | 8.1 |  | (3.8-16.2) | 5.7 |  | (0.9-13.7) |  | 7.4 |  | (3.4-13.4) | |
| *temra* | 0.4 | |  | (0.1-8.7) |  | 0.3 |  | (0.0-1.2) | 0.3 |  | (0.0-7.4) |  | 0.3 |  | (0.0-1.5) | |
|  |  | |  |  |  |  |  |  |  |  |  |  |  |  |  | |
| *CD8* |  | |  |  |  |  |  |  |  |  |  |  |  |  |  | |
| *naive* | 44.7 | |  | (16-76.9) |  | 28.8 |  | (8.0-64.0) | 55.5 |  | (23.9-79) |  | 34.4 |  | (12.3-68.2) | |
| *cm* | 17.0 | |  | (2.6-30.5) |  | 22.0 |  | (10.2-55.2) | 15.9 |  | (5.9-33.3) |  | 20.6 |  | (9.6-47.2) | |
| *em* | 6.9 | |  | (0.0-23.9) |  | 10.6 |  | (4.2-29.6) | 5.8 |  | (0.0-17.7) |  | 9.0 |  | (2.2-21.1) | |
| *temra* | 27.5 | |  | (10.9-62.6) |  | 30.4 |  | (12.7-61) | 20.9 |  | (8.9-53.0) |  | 28.3 |  | (9.0-62.4) | |
|  |  | |  |  |  |  |  |  |  |  |  |  |  |  |  | |
| *γδ T cells* |  | |  |  |  |  |  |  |  |  |  |  |  |  |  | |
| *naïve-like* | 8.4 | |  | (2.3-61.2) |  | 5.9 |  | (1.6-31) | 12.9 |  | (2.4-65.1) |  | 9.8 |  | (2.2-52.5) | |
| *cm-like* | 49.1 | |  | (0.2-82.2) |  | 38.3 |  | (11.2-74.1) | 53.3 |  | (0.5-75.7) |  | 43.2 |  | (5.6-78.5) | |
| *em-like* | 24.3 | |  | (0.4-55.5) |  | 24.3 |  | (5.6-60.8) | 19.5 |  | (0.6-50.5) |  | 18.7 |  | (5.6-72.2) | |
| *temra-like* | 8.2 | |  | (1.7-70.8) |  | 15.5 |  | (1.6-76.5) | 7.2 |  | (0.9-58.5) |  | 17.0 |  | (0.8-76.9) | |
|  | N=52 | | | |  | N=72 | | | N=60 | | |  | N=60 | | | |

**Supplementary Table 7: Frequencies of lymphocyte subsets including memory values in healthy adults.** Frequencies of CD4conv, Tregs, CD8^+^CD4^+^, CD8^+^, γδ T cells, B cells, NK cells and NKT cells are expressed as percentage of live lymphocytes. Frequencies of Vγ9^+^, Vγ9^-^, CD20^+^, and CD20^-^ are expressed as percentage of respective parent gate. Memory subpopulation frequencies are shown as percentage of respective parent gate. Data are presented as median (2.5%-97.5%) percentile. All frequency reference values originate from panel 2.

|  | | Males | | | | | | | Females | | | | | | |  |
| --- | --- | --- | --- | --- | --- | --- | --- | --- | --- | --- | --- | --- | --- | --- | --- | --- |
|  | 18-40 years old | | | |  | 41-69 years old | | | 18-40 years old | | |  | 41-69 years old | | | |
| *LEU (x103/µl)* | 5.8 | |  | (4-9.8) |  | 5.9 |  | (3.5-10.1) | 6.7 |  | (4.2-10.7) |  | 6.1 |  | (4.1-9.7) | |
| *LY (x103/µl)* | 1.8 | |  | (1.1-2.8) |  | 1.5 |  | (0.9-2.8) | 2.0 |  | (1.1-3.1) |  | 1.5 |  | (0.9-2.7) | |
| *Hb (g/dl)* | 15.2 | |  | (13.4-16.8) |  | 15.0 |  | (13.4-17.1) | 13.5 |  | (11.8-15.4) |  | 13.5 |  | (11.9-15.2) | |
| *THR (x103/µl)* | 246.6 | |  | (168.2-368.4) |  | 259.3 |  | (202.6-363.6) | 272.5 |  | (134.6-382.3) |  | 290.7 |  | (215.2-441.8) | |
|  |  | |  | N=52 | |  |  | N=72 |  |  | N=60 |  |  |  | N=60 | |
|  | | | | | | | | | | | | | | | | |

**Supplementary Table 8: Complete Blood Count values** **in healthy adults**. Data are presented as median (2.5%-97.5%) percentile. LEU: Leukocytes; LY: Lymphocytes; Hb: Hemoglobin; THR: Thrombocytes.
