# Supplementary figures and images for: Distribution of major lymphocyte subsets and memory T-cell subpopulations in healthy adults employing GLP-conforming multicolor flow cytometry
Source: Leukemia. 2021 Jul 21;35(10):3021–5. doi: 10.1038/s41375-021-01348-5 (PMC8478656; doi:10.1038/s41375-021-01348-5)

Supplementary Figure 1

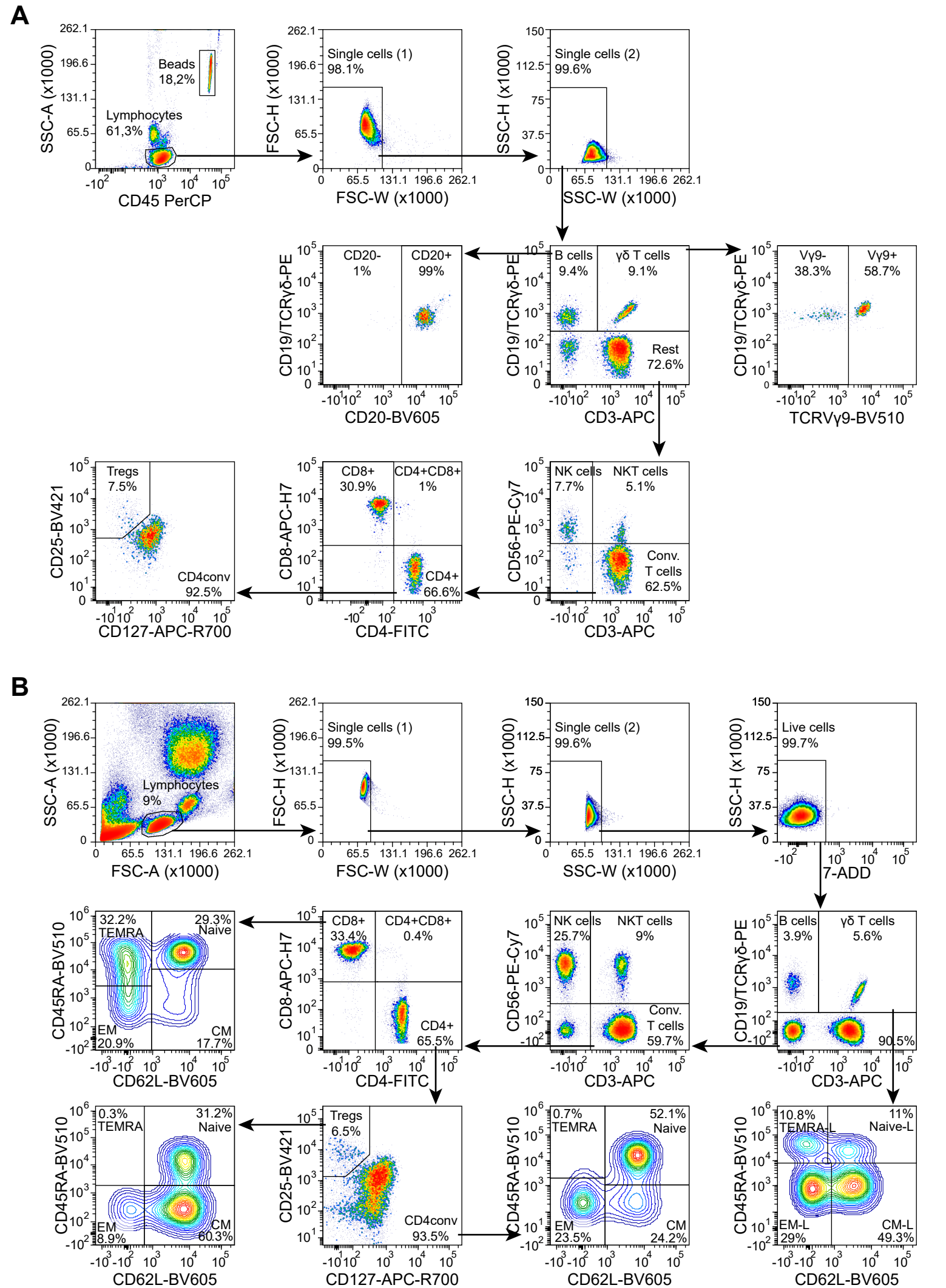

Supplement: Supplementary file 2 — Supplementary Figure 1 [file 41375_2021_1348_MOESM2_ESM.pdf]

Supplementary Figure 2

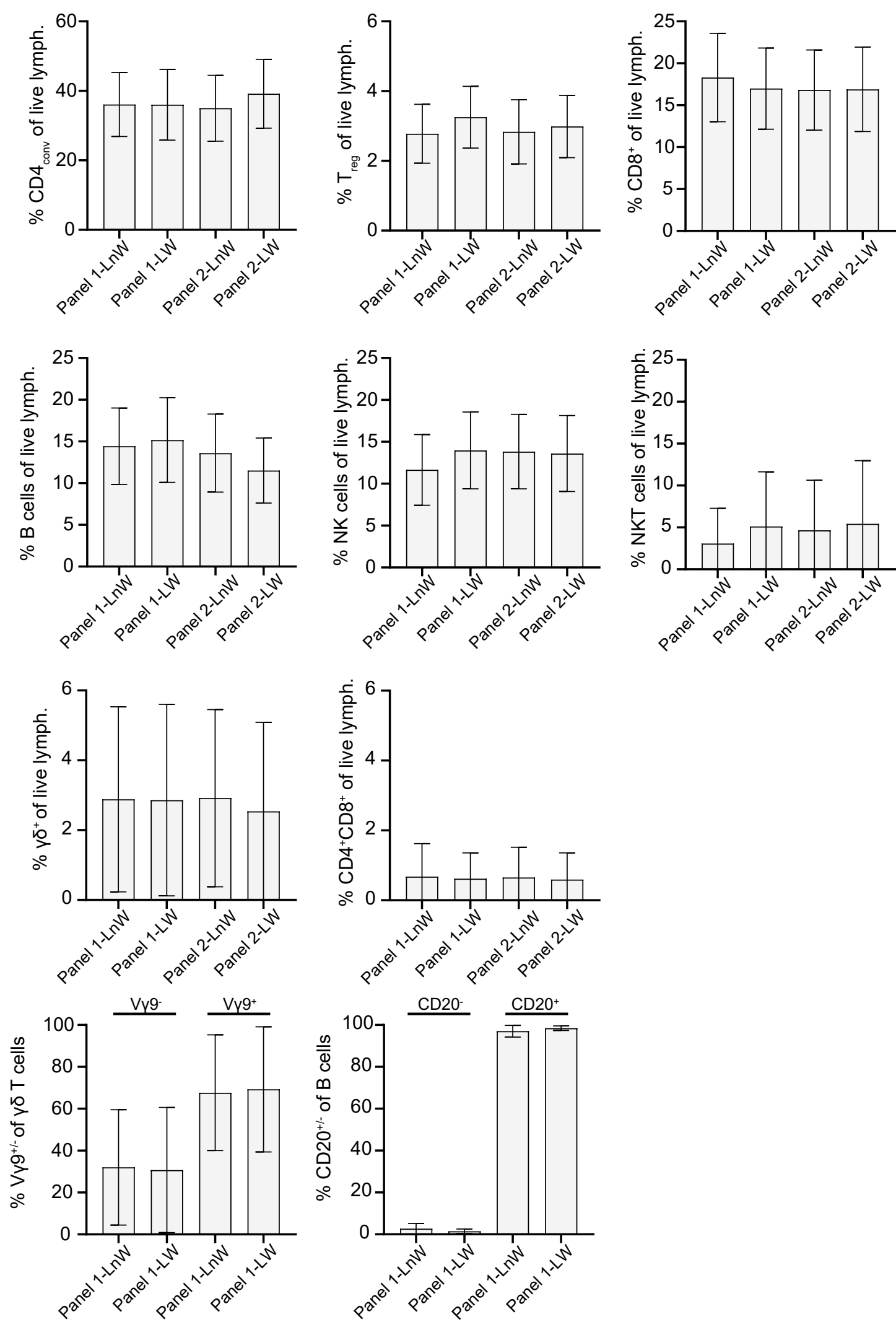

Supplement: Supplementary file 3 — Supplementary Figure 2 [file 41375_2021_1348_MOESM3_ESM.pdf]

Supplementary Figure 3

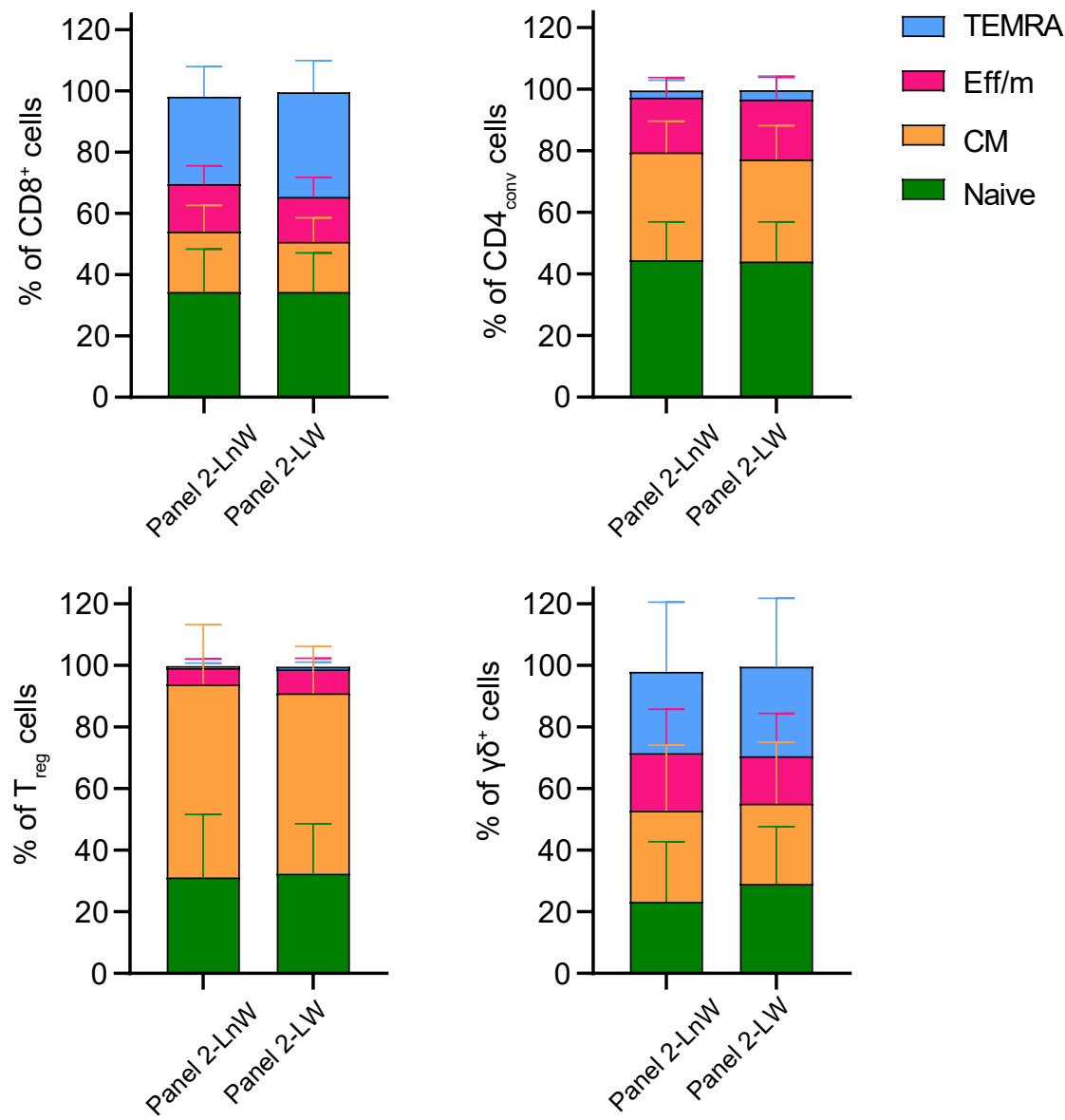

Supplement: Supplementary file 4 — Supplementary Figure 3 [file 41375_2021_1348_MOESM4_ESM.pdf]

# Supplementary Figure 4

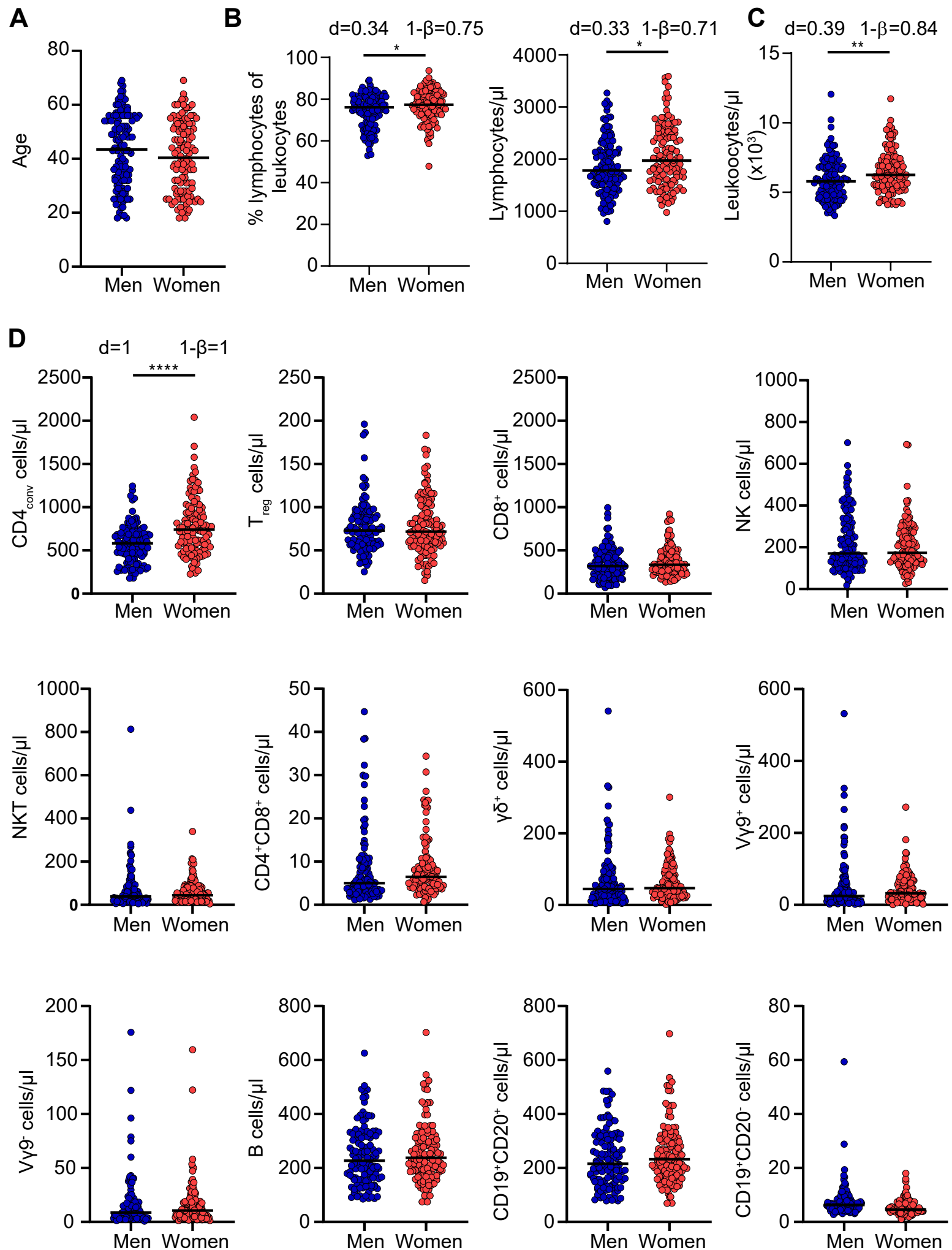

Supplement: Supplementary file 5 — Supplementary Figure 4 [file 41375_2021_1348_MOESM5_ESM.pdf]

Supplementary Figure 5

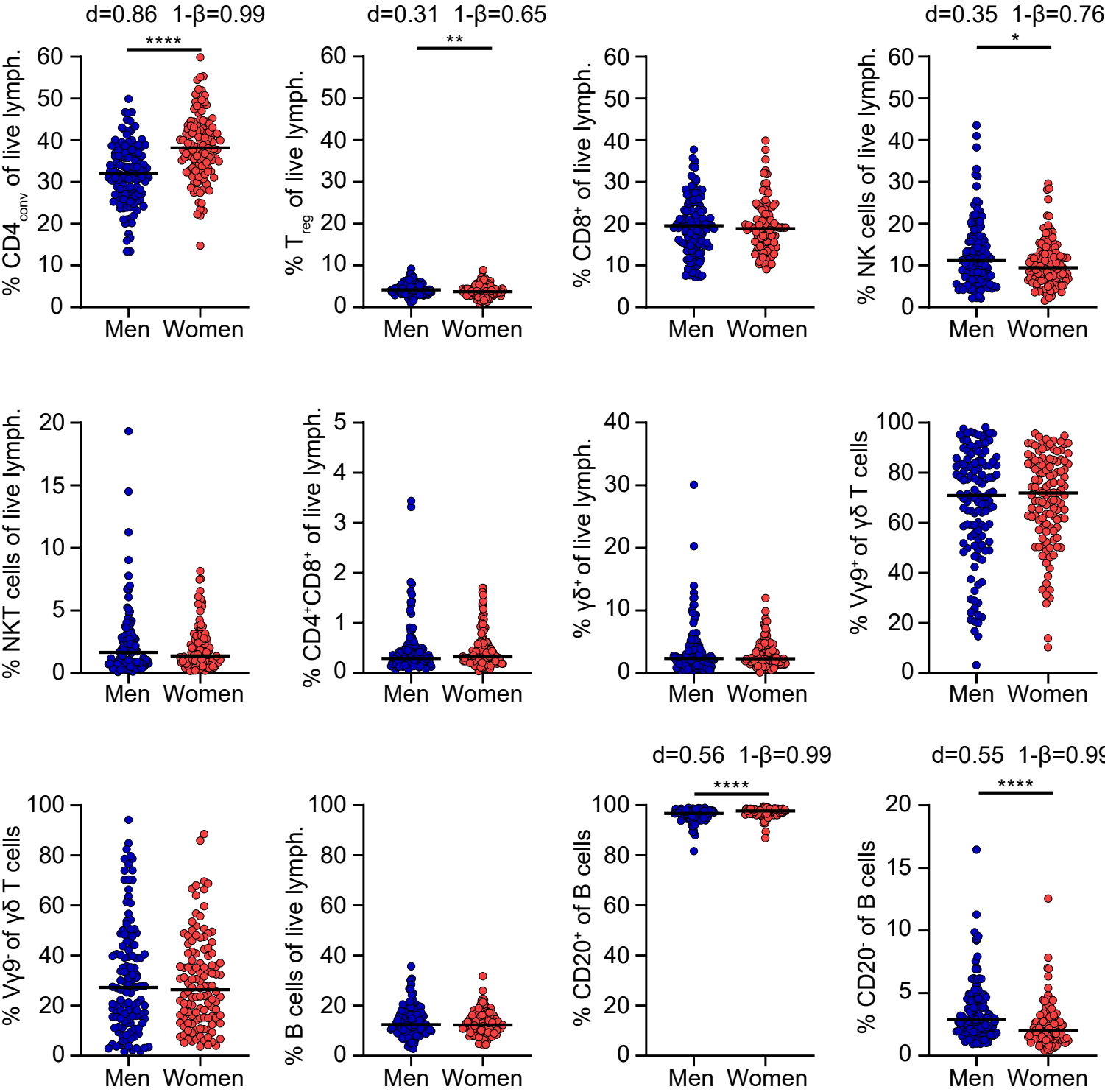

Supplement: Supplementary file 6 — Supplementary Figure 5 [file 41375_2021_1348_MOESM6_ESM.pdf]

# Supplementary Figure 6

**A**

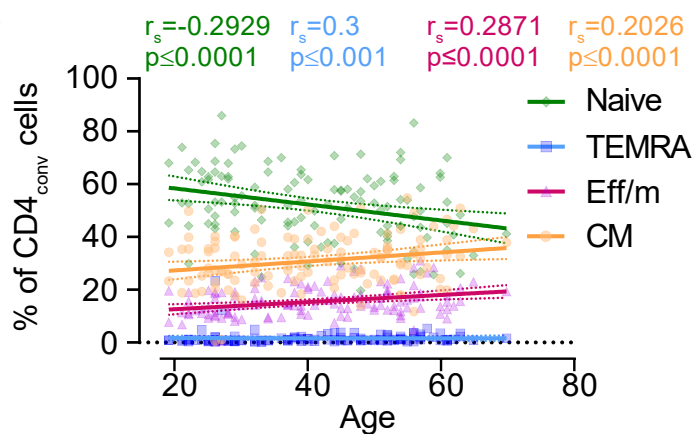

**B**

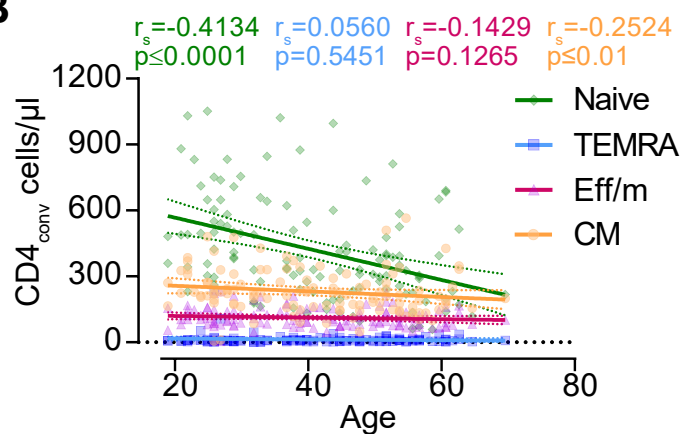

**C**

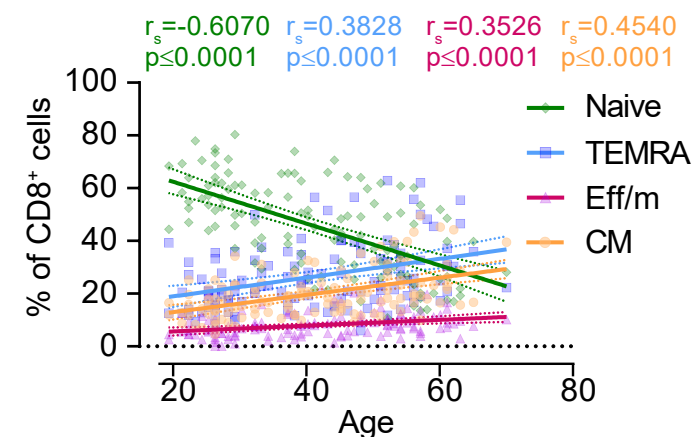

**D**

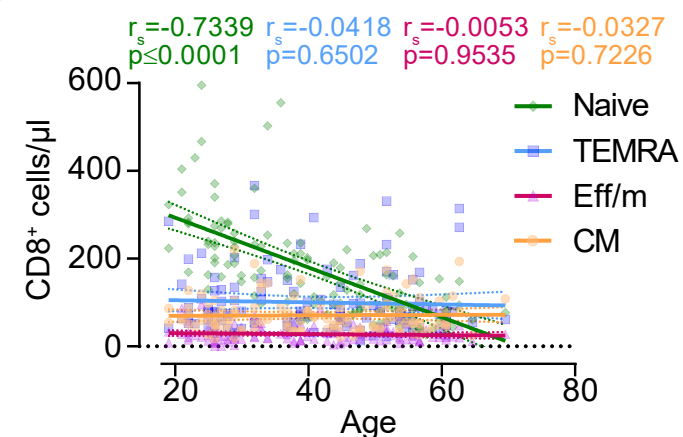

**E**

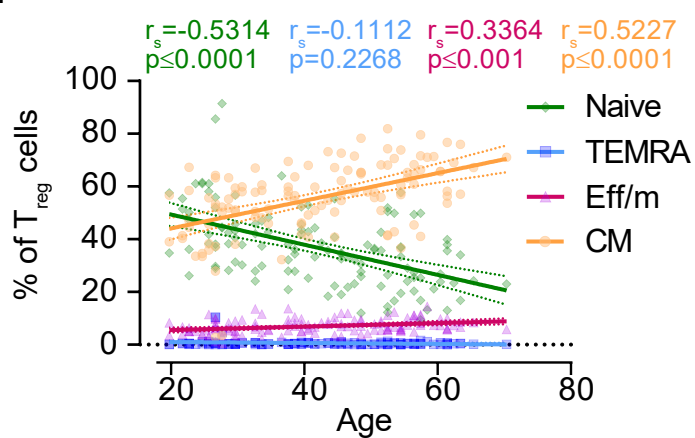

**F**

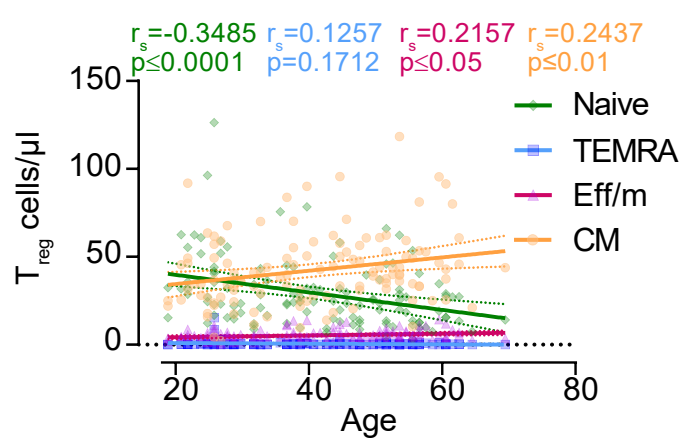

**G**

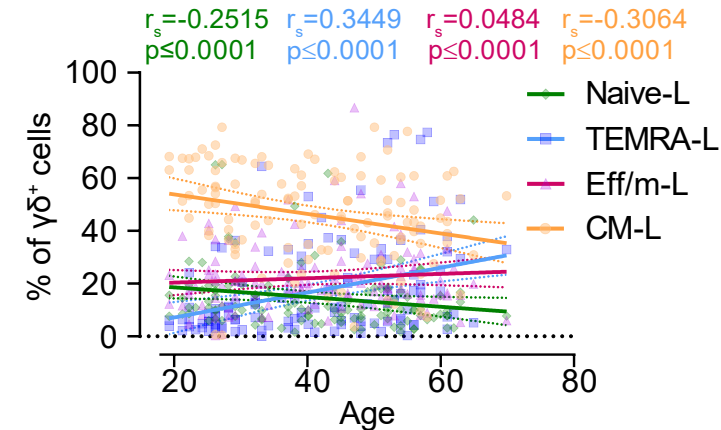

**H**

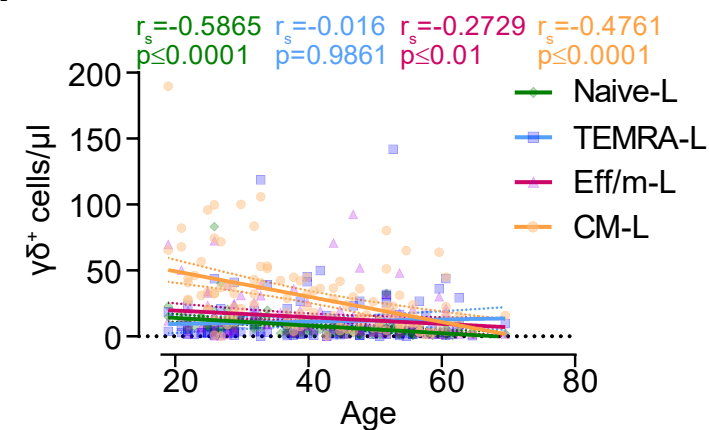

Supplement: Supplementary file 7 — Supplementary Figure 6 [file 41375_2021_1348_MOESM7_ESM.pdf]

# Supplementary Figure 7

**A**

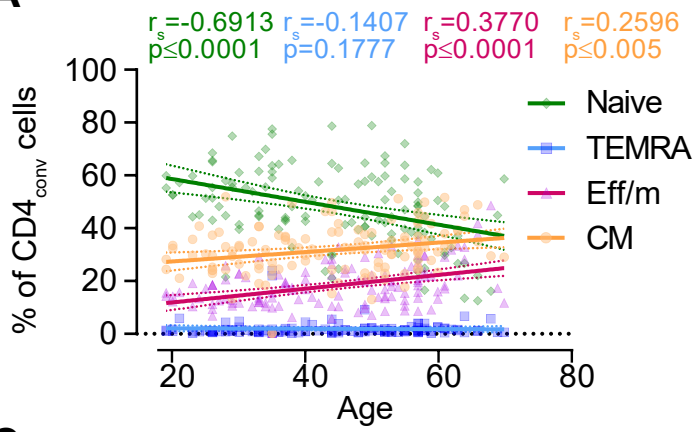

**B**

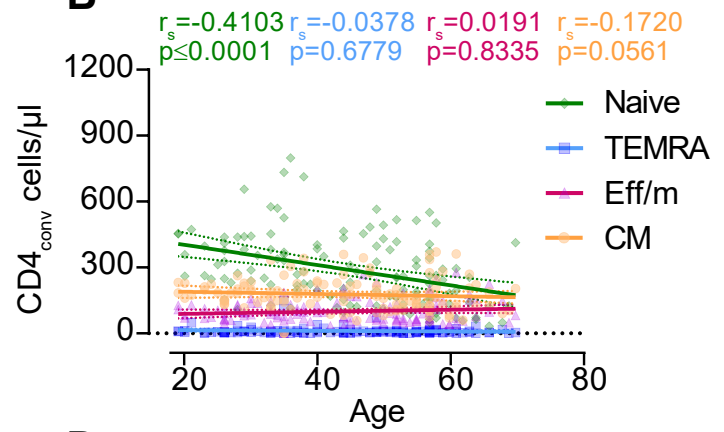

**C**

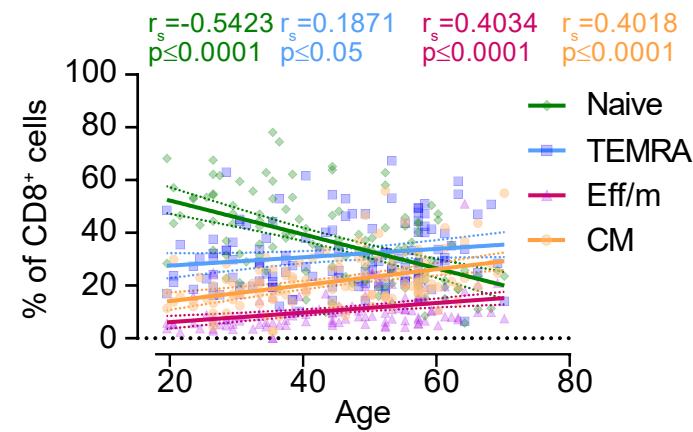

**D**

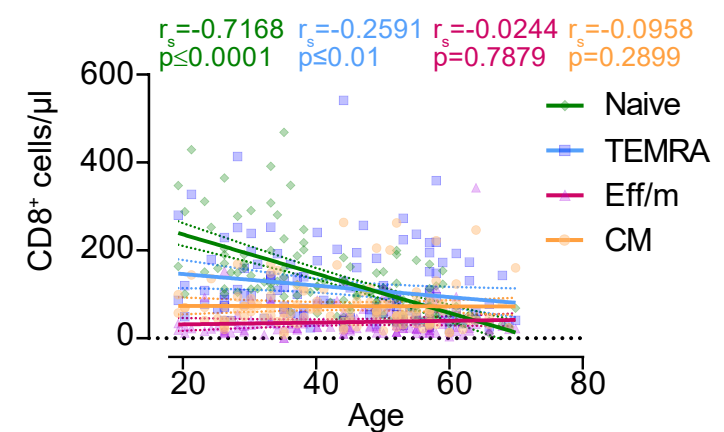

**E**

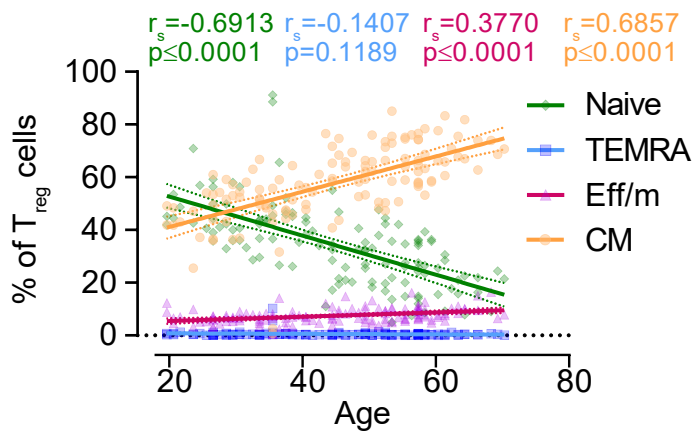

**F**

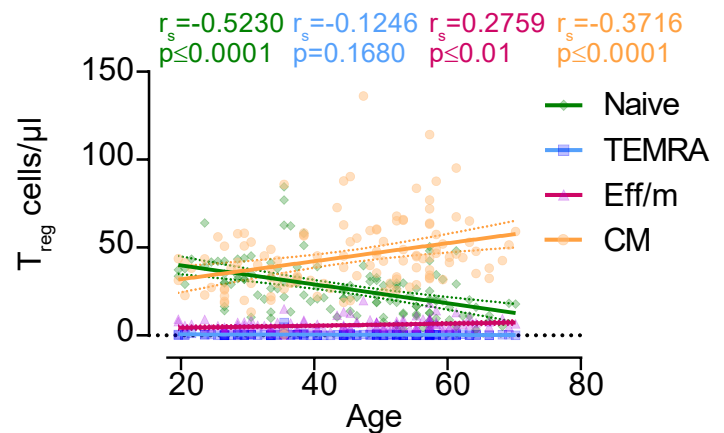

**G**

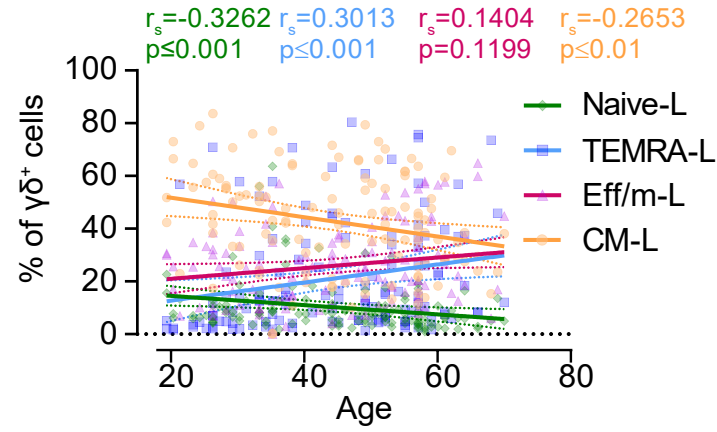

**H**

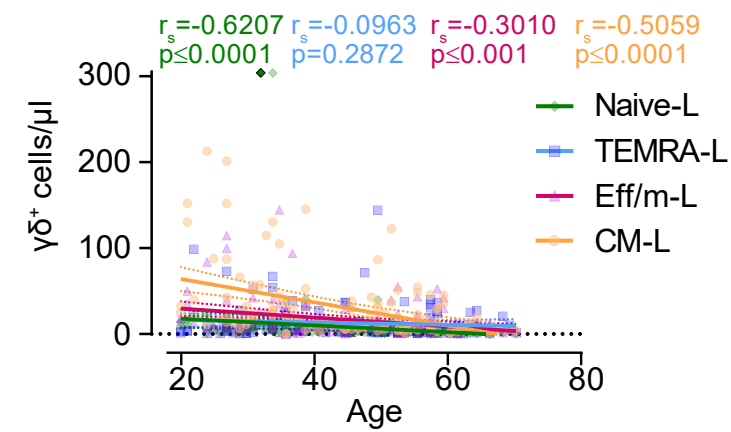

Supplement: Supplementary file 8 — Supplementary Figure 7 [file 41375_2021_1348_MOESM8_ESM.pdf]
